# Autoencoder-Based Representation Learning for Similar Patients Retrieval From Electronic Health Records: Comparative Study

**DOI:** 10.2196/68830

**Published:** 2025-07-24

**Authors:** Deyi Li, Aditi Shukla, Sravani Chandaka, Bradley Taylor, Jie Xu, Mei Liu

**Affiliations:** 1 Department of Health Outcomes & Biomedical Informatics, University of Florida, 1889 Museum Rd, 7th Floor, Suite 7000, Room 7012, Gainesville, FL, 32611, United States, 1 352-627-9143; 2 Department of Mathematics, College of Arts and Sciences, University of Pennsylvania, Philadelphia, PA, United States; 3 Department of Population Health, University of Kansas Medical Center, Kansas City, KS, United States; 4 CTSI Center for Biomedical Informatics, Medical College of Wisconsin, Milwaukee, WI, United States

**Keywords:** machine learning, decision support for health professionals, methods and instruments in medical informatics, electronic health records

## Abstract

**Background:**

By analyzing electronic health record snapshots of similar patients, physicians can proactively predict disease onsets, customize treatment plans, and anticipate patient-specific trajectories. However, the modeling of electronic health record data is inherently challenging due to its high dimensionality, mixed feature types, noise, bias, and sparsity. Patient representation learning using autoencoders (AEs) presents promising opportunities to address these challenges. A critical question remains: how do different AE designs and distance measures impact the quality of retrieved similar patient cohorts?

**Objective:**

This study aims to evaluate the performance of 5 common AE variants—vanilla autoencoder, denoising autoencoder, contractive autoencoder, sparse autoencoder, and robust autoencoder—in retrieving similar patients. Additionally, it investigates the impact of different distance measures and hyperparameter configurations on model performance.

**Methods:**

We tested the 5 AE variants on 2 real-world datasets—the University of Kansas Medical Center (n=13,752) and the Medical College of Wisconsin (n=9568)—across 168 different hyperparameter configurations. To retrieve similar patients based on the AE-produced latent representations, we applied k-nearest neighbors (k-NN) using Euclidean and Mahalanobis distances. Two prediction targets were evaluated: acute kidney injury onset and postdischarge 1-year mortality.

**Results:**

Our findings demonstrate that (1) denoising autoencoders outperformed other AE variants when paired with Euclidean distance (*P*<.001), followed by vanilla autoencoders and contractive autoencoders; (2) learning rates significantly influenced the performance of AE variants; and (3) Mahalanobis distance-based k-NN frequently outperformed Euclidean distance-based k-NN when applied to latent representations. However, whether AE models are superior in transforming raw data into latent representations, compared with applying Mahalanobis distance-based k-NN directly to raw data, appears to be data-dependent.

**Conclusions:**

This study provides a comprehensive analysis of the performance of different AE variants in retrieving similar patients and evaluates the impact of various hyperparameter configurations on model performance. The findings lay the groundwork for future development of AE-based patient similarity estimation and personalized medicine.

## Introduction

Diseases vary in complexity, posing substantial challenges in diagnosis, treatment, and prognosis—even when cases appear clinically similar [1]. This heterogeneity is particularly prominent in complex disorders like autoimmune diseases [2], Parkinson disease [3], and cardiovascular diseases [4], where underlying causes often result from a confluence of genetic, environmental, and lifestyle factors [5]. As these complexities become more evident, the rapid adoption of electronic health record (EHR) systems has bolstered the potential for personalized medicine to enhance patient care. Personalized medicine focuses on tailoring treatments and predicting patient outcomes by analyzing data from patients with similar characteristics [6]. By assessing EHR snapshots of comparable patients—including prescriptions, procedures, vital signs, lab results, and clinical outcomes—physicians can proactively predict disease onsets, customize treatment plans, and anticipate patient-specific trajectories [7,8]. Additionally, predictive models that leverage data from similar patients tend to be more accurate, as they capture localized data patterns that might be obscured in aggregated data [9].

Retrieving a high-quality set of similar patients is central to personalized medicine, directly impacting both evidence-based decision-making and the accuracy of personalized predictive models. However, EHR data are inherently challenging to model due to high dimensionality, mixed feature types, noise, bias, and sparsity, complicating the effective retrieval of similar patients [10]. For instance, applying traditional Euclidean distance-based k-nearest neighbors (k-NN) directly to EHR data may be problematic due to high dimensionality and mixed data types. To address these challenges, various similar patient retrieval algorithms have been proposed, incorporating advanced feature engineering to handle mixed features and reduce dimensionality [11-13].

Patient representation learning offers new avenues for overcoming these obstacles, with autoencoders (AEs) being one of the most important and widely used methods in this area [14-17]. AEs compress input data into a lower-dimensional latent space, known as a latent representation, and reconstruct it back to its original form, facilitating effective auto-feature engineering and patient representation [18,19]. AEs are particularly useful for encoding nonlinear relationships within EHR, and capturing complex structures in clinical data [20]. As AE applications to EHR increase, their use is becoming increasingly diverse [14-16]. For instance, Chowdhury et al [16] designed a mixed pooling multi-view attention AE to learn representations that encapsulate a holistic view of patient medical profiles. Beaulieu-Jones et al [15] applied a vanilla AE with a modified binary cross-entropy loss to impute missing data in EHR, and Lee et al [14] used a dual adversarial AE to generate sequential EHR data.

In personalized medicine, AEs are increasingly applied to enhance similar patient retrieval [21-23]. Generally, these studies use AEs to generate efficient patient representations from EHR data, with similarity among patients assessed using distance measures such as Euclidean and Mahalanobis distances. For example, Jo et al [21] used a supervised AE to incorporate disease labels into latent representations and calculated patient similarity in the latent space using the Euclidean distance. Miotto et al [22] introduced the “Deep Patient” framework with a 3-layer stack of denoising autoencoders (DAEs) to generate latent patient representations from EHR data, which was then used to estimate patient similarity. Landi et al [23] used a convolutional AE to transform patient trajectories into low-dimensional latent vectors and achieved patient risk stratification by patient similarity. These studies underscore the potential of AEs to drive advances in personalized medicine.

Despite the promising results of applying AEs to EHR data, a critical question remains unanswered: how do different AE designs impact performance in similar patient retrieval tasks? Existing studies have not clearly justified their choices of specific AE designs. Specifically, AE designs encompass 2 key aspects. The first aspect is the choice of the base AE model, as different AE variants may perform differently due to their distinct design focuses and the unique characteristics of EHR data. When these base AE models are integrated into more complex architectures (eg, “Deep Patient” [22]), their behavior may also vary. Therefore, gaining deeper insight into the performance of different base AE models on EHR data is valuable. The second aspect is hyperparameter tuning, as AEs are known to be highly sensitive to hyperparameters, including learning rate, latent dimensionality, and optimization techniques [24]. Therefore, understanding how different hyperparameters impact AE performance on EHR data is also important.

In this study, we used 2 real-world EHR datasets from the University of Kansas Medical Center (KUMC) and the Medical College of Wisconsin (MCW), covering the period from January 1, 2016 to December 31, 2016, to evaluate the performance of 5 widely used AE variants for retrieving similar patients: vanilla AE (AE) [18], DAE [25], contractive autoencoder (CAE) [26], sparse autoencoder (SAE) [27], and robust autoencoder (RAE) [28]. Vanilla AE is the most basic form of autoencoder, making it efficient to train and use. DAE and RAE can address the significant noise in EHR data, while CAE and SAE use different mechanisms to learn more robust latent representations for EHR data with complex distributions [29]. Additionally, we investigated the impact of 2 distance measures, Euclidean and Mahalanobis, on similar patient retrieval when paired with these AE variants. To comprehensively evaluate model performance, we tested them within a standard k-NN classification framework for 2 binary clinical outcomes in hospitalized patients: acute kidney injury (AKI) onset and 1-year mortality postdischarge, representing short-term disease and long-term survival risk classification scenarios. AKI, a life-threatening and heterogeneous condition prevalent among hospitalized patients, is particularly suited to a personalized approach. Finally, we explored how different hyperparameter configurations affect AE performance in retrieving similar patients for outcome prediction. This study provides key insights into AE optimization for personalized medicine applications, informing future advancements in EHR-driven patient care.

## Methods

### Comparison Framework Overview

This study aims to evaluate the effects of various AE variants, hyperparameter settings, and distance measures on the performance of similar patient retrieval. The 5 AE variants investigated were vanilla AE (AE), DAE, CAE, SAE, and RAE. Each AE was trained in an unsupervised manner on the training dataset, after which both the training and test datasets were transformed into latent representations using the trained AEs.

Performance was evaluated using a standard k-NN classification framework with neighborhood sizes of 5, 10, 15, and 20. For similar patient retrieval, Euclidean and Mahalanobis distances were applied to the latent representations to identify similar patients based on a specified neighborhood size for each test patient. Labels were assigned to each test patient through majority voting, and these assigned labels were then compared with the ground truth to assess model accuracy. Furthermore, we analyzed the influence of different hyperparameter configurations on AE model performance in retrieving similar patients, focusing on Euclidean distance as the patient similarity measure.

### Data Source and Processing

Our primary dataset consisted of inpatient data extracted from KUMC, covering admissions from January 1, 2016, to December 31, 2016. To assess the generalizability of our findings, we extracted an external validation dataset from MCW for the same period.

Both datasets were processed using the same protocol. The inclusion criteria were as follows: (1) older than 18 years, (2) baseline serum creatinine (SCr) <3.5 mg/dL, and (3) AKI onset occurring at least 72 hours postadmission to focus only on hospital-acquired AKI [30]. AKI was defined using the SCr criteria described in the “Kidney Disease: Improving Global Outcomes” clinical practice guidelines [31]. For patients with multiple admissions, only the first encounter was retained. The study focused on 3 types of in-hospital clinical features: medications, procedures, and lab test results. The data observation window for these features extended from 48 hours before the prediction point up to the prediction point. For patients with AKI, the prediction point was set at 24 hours before AKI onset, while for patients without AKI, it was set at 24 hours before the last SCr measurement [9].

Medications were represented by the maximum dosages recorded within the data observation window, procedures were encoded as binary values indicating whether a procedure was performed during the observation window, and lab test results were recorded as the most recent values within the observation window. Medications and procedures present in less than 1% of patients were excluded from the analysis. Lab tests with a missing rate over 30% were also discarded, with the remaining missing lab values imputed using the multiple imputation by chained equations method [32]. Outliers were replaced using the Winsorizing method with a 1% threshold [33], and min-max normalization was applied to scale values between 0 and 1.

In addition to AKI onset, 1-year mortality after discharge was also included as a prediction target, providing a comprehensive evaluation of the retrieved similar patient cohorts in terms of both short-term (AKI onset) and long-term (1-year mortality) clinical outcomes.

### AE Variants

**Figure 1.**
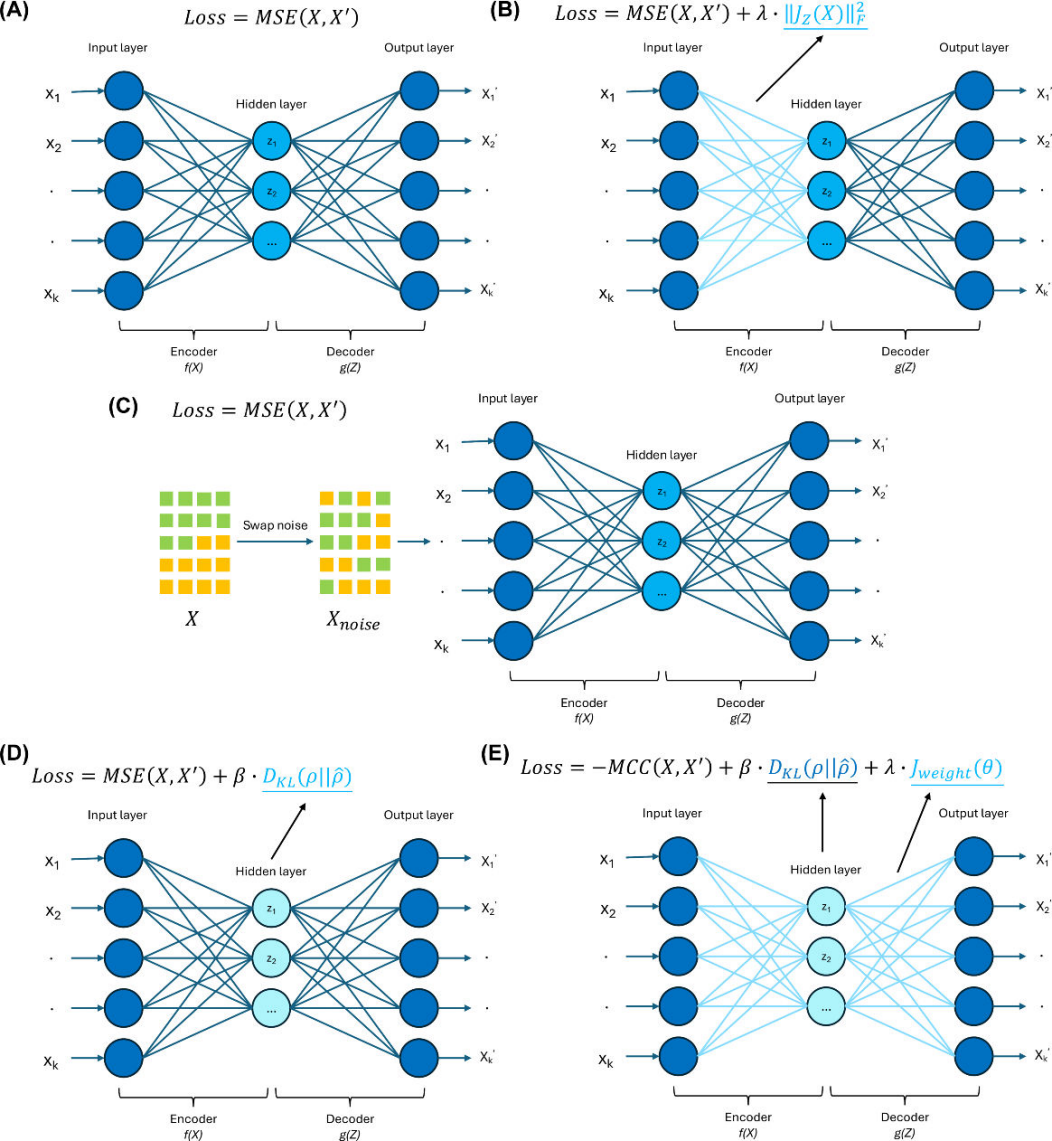
Overview of the 5 autoencoder (AE) variant designs with different loss functions. **(A)** Vanilla AE; **(B)** Contractive AE; **(C) **Denoising AE; **(D) **Sparse AE; and **(**E**) **Robust AE. MCC: maximum correntropy criterion; MSE: mean squared error.

The vanilla AE (Figure 1A) is the most basic form in the autoencoder family. Its architecture is a symmetric feedforward neural network structure, though this symmetry does not necessarily apply to the weights, biases, or activation functions. It has 2 main components: an encoder and a decoder. The encoder encodes the input into a latent representation, while the decoder reconstructs the original data from this hidden representation. For an AE with a single hidden layer, the input data undergo the following transformations:


$$Z=f\left(X\right)=\phi_{1}(WX+b)$$

$$X^{`}=g\left(Z\right)=\phi_{2}(W^{`}Z+b`)$$

Here, X denotes the input data, and X` represents the reconstructed data. Z is the output of the latent representation produced by the encoder. W, W`, b, and b` are the weights and biases of the encoder and decoder, while $\phi_{1}$ and $\phi_{2}$ are activation functions. To quantify reconstruction accuracy, the mean squared error (MSE) loss was used, which measures the difference between the original input data and the reconstructed data, as follows:


$${Loss}_{AE}=MSE(X,X`)=\frac{1}{n}\sum_{i=1}^{n}{\left(x_{i}-{x`}_{i}\right)}^{2}$$

Here, n denotes the number of input samples, while $x_{i}$ and ${x`}_{i}$ represent the i-th sample of X and the i-th sample of X`, respectively. This loss function allows AE to learn a compressed representation of the data by minimizing the reconstruction error.

The DAE (Figure 1C) enhances model robustness by introducing noise into the input data. Specifically, data with noise ($X_{noise}$ in Figure 1C) are used as input, and the reconstruction error is calculated between the original noise-free data (X in Figure 1C) and the reconstructed data. In this way, the model learns to reconstruct the input by eliminating any noise present. In this study, we used swap noise, where each value in the training data may be replaced with a random value from the same column with a certain probability. We selected swap noise for fairness purposes as it has been shown to be the most effective noise type for tabular data [34]. Other commonly used noise types include Gaussian noise and masking noise [35].

The CAE (Figure 1B) enhances model robustness by reducing the encoder’s sensitivity to minor perturbations in the input, a typical vulnerability in AEs where small variations can lead to significant differences in latent representations. The CAE addresses this issue by introducing an additional penalty term, the Frobenius norm of the encoder, to the loss function. This term, which is the L2-norm of the Jacobian matrix of the hidden layer, makes the encoder output more stable against small input variations. The Frobenius norm of the encoder and the CAE loss function are shown as follows:


$${\left\|J_{Z}(X)\right\|}_{F}^{2}=\frac{1}{n}\sum_{i=1}^{n}\sum_{j=1}^{m}\sum_{k=1}^{l}{\left(\frac{\partial z_{i}^{\left(k\right)}(x_{i})}{\partial x_{i}^{\left(j\right)}}\right)}^{2}$$

$${Loss}_{CAE}=MSE\left(X,X^{`}\right)+{\lambda ∙\left\|J_{Z}(X)\right\|}_{F}^{2}$$

Here, m denotes the number of input features, and l represents the length of the latent representations. λ controls the strength of the additional penalty term, which aims to restrict the rate of change in the encoder output relative to changes in the input. When the input undergoes minor variations, the CAE encoder output remains relatively stable, enhancing the model’s robustness against noise and small perturbations.

The SAE (Figure 1D) encourages the model to learn efficient representations by enforcing sparsity in latent representations. By adding a Kullback-Leibler (KL) divergence penalty between a Bernoulli distribution and the distribution of latent layer outputs to the loss function, the SAE limits the number of active neurons (whose outputs are significantly nonzero) in the latent layer. This helps SAE to capture key information from the input using a limited number of active neurons in the latent layer, preventing it from simply copying the input to the output and enhancing the model’s ability to capture the inherent structure of the input data. The additional penalty term and the loss function of the SAE are as follows:


$$D_{KL}(\rho \vee \left|\hat{\rho }\right)=\rho log\frac{\rho }{\hat{\rho }}+(1-\rho )log\frac{\left(1-\rho \right)}{\left(1-\hat{\rho }\right)}$$

$${Loss}_{SAE}=MSE\left(X,X^{`}\right)+\beta ∙D_{KL}(\rho \vee \left|\hat{\rho }\right)$$

Here, ρ denotes the mean of the Bernoulli distribution. $\hat{\rho }$ denotes the mean of the distribution of latent representations over the training data. β controls the strength of the additional penalty term.

The RAE (Figure 1E) improves noise tolerance by using the maximum correntropy criterion (MCC) instead of MSE for reconstruction error, making it less sensitive to outliers [36]. The intuition behind the MCC-based reconstruction error is that as the distance between X and X` increases, the corresponding measure transitions from the L2 norm to the L1 norm, and eventually to the zero norm when X and X` are far apart. The RAE also includes a sparsity penalty term similar to that of the SAE, along with an additional weight decay term to prevent overfitting. The MCC-based reconstruction error, the weight decay term, and the final loss function of the RAE are shown as follows:


$$MCC(X,X`)=\frac{1}{n}\sum_{i=1}^{n}\sum_{j=1}^{m}\frac{1}{\sqrt{2\pi }\sigma }exp(-\frac{{\left(x_{i}^{k}-{x`}_{i}^{\left(j\right)}\right)}^{2}}{2\sigma^{2}})$$

$$J_{weight}\left(\theta \right)=\frac{1}{2}\sum_{L=1}^{2}\sum_{i=1}^{s_{L}}\sum_{j=1}^{s_{L+1}}{{(w}_{ji}^{\left(L\right)})}^{2}$$

$${Loss}_{RAE}=-MCC\left(X,X^{`}\right)+{\beta ∙D}_{KL}(\rho \vee \left|\hat{\rho }\right)+\lambda ∙J_{weight}\left(\theta \right)$$

Here, σ denotes the variance of the Gaussian distribution. $w_{ji}^{\left(L\right)}$ denotes an element in the weight matrix of the L-th layer. $S_{L}$ denotes the number of neurons in the L-th layer. β and λ control the strength of the 2 penalty terms, respectively.

### Patient Similarity Measures

To identify similar patients, we applied 2 distance measures—Euclidean and Mahalanobis distances—to the latent representations generated by each AE variant. For each patient in the test dataset, we used these distance measures to find a cohort of similar patients from the training dataset. The Euclidean distance on the latent representations is calculated as follows:


$$D_{Euclidean}(z_{i},z_{j})=\sqrt{\sum_{k=1}^{l}{\left(z_{i}^{k}-z_{j}^{k}\right)}^{2}}$$

Here, $z_{i}$ denotes the i-th patient in the training set and $z_{j}$ denotes the j-th patient in the test set. l denotes the length of the latent representations.

The Mahalanobis distance can be viewed as a Euclidean distance after applying a linear transformation to the feature space, defined by L:


$$D_{Mahalanobis}(z_{i},z_{j})=\sqrt{{\left(Lz_{i}-Lz_{j}\right)}^{T}(Lz_{i}-Lz_{j})}$$

In this study, we used 3 different algorithms to estimate the Mahalanobis distance on the latent representations: large margin nearest neighbor (LMNN) [37], Neighborhood Components Analysis (NCA) [38], and Metric Learning for Kernel Regression (MLKR) [39].

- LMNN learns a Mahalanobis distance within the standard k-NN classification framework, aiming to bring the nearest *k* neighbors from the same class closer while ensuring that examples from different classes are separated by a large margin.
- NCA enhances the accuracy of nearest neighbor classification compared with the traditional Euclidean distance by directly maximizing a stochastic version of the leave-one-out k*-*NN score on the training set.
- MLKR learns a Mahalanobis distance by directly minimizing the leave-one-out regression error. This algorithm can also be viewed as a supervised extension of principal component analysis (PCA), making it suitable for dimensionality reduction and visualization of high-dimensional data.

### Experimental Design

**Figure 2.**
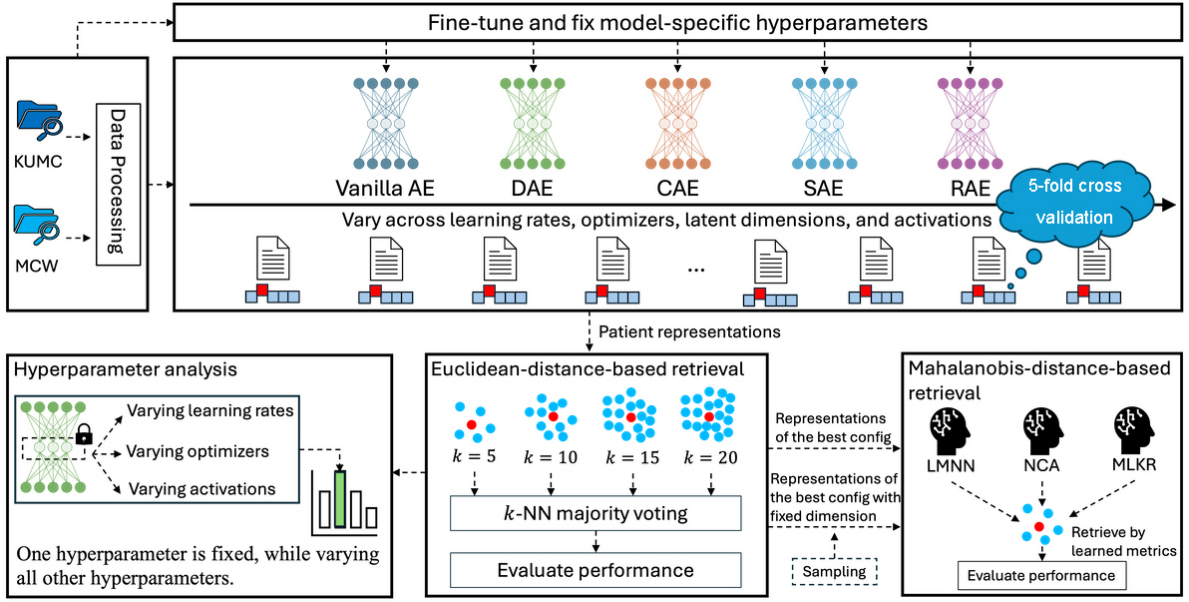
Workflow of the study. After the electronic health record data were transformed into latent representations using 5-fold cross-validation, the latent representations were used for 3 downstream evaluations: Euclidean-distance-based similar patient retrieval, Mahalanobis-distance-based similar patient retrieval, and AE hyperparameter analysis. AE: autoencoder; CAE: contractive autoencoder; DAE: denoising autoencoder; KUMC: University of Kansas Medical Center; LMNN: large margin nearest neighbor; MCW: Medical College of Wisconsin; MLKR: metric learning for kernel regression; NCA: neighborhood components analysis; RAE: robust autoencoder; SAE: sparse autoencoder.

The workflow of the study is presented in Figure 2. Each AE variant in this study consisted of a 3-layer structure: an input layer, a hidden layer, and an output layer. Previous research suggests that additional hidden layers in AEs do not necessarily lead to improved downstream task performance [40]. All AE variants were implemented in PyTorch (version 2.4.0; Meta AI), and trained on 2 NVIDIA GeForce RTX 2080 Ti GPUs, each with 10.7 GB of RAM. For each of the 5 AE variants, we performed an exhaustive grid search to explore all possible combinations of learning rates, optimizers, latent dimensions, and activation functions, resulting in 168 different hyperparameter configurations per AE variant. Details of the hyperparameter space are listed in Table 1. The Mahalanobis distance was estimated using the metric-learn library [41].

**Table 1.** Hyperparameter configuration space. The combinations of different values for the 4 studied hyperparameters resulted in 168 different hyperparameter configurations.

Hyperparameter	Range
Learning rate	1E-5, 1E-4, 1E-3, and 1E-2
Optimizer	Adam, Adamax, and RMSprop
Latent dimension : input dimension	0.02, 0.05, 0.10, 0.15, 0.30, 0.50, and 0.75
Activation functions	Sigmoid and rectified linear unit (ReLU)

To assess the generalizability of our findings, we applied the following steps in parallel to both the KUMC and MCW datasets. Before evaluating the performance of different AE variants on the 4 hyperparameters of interest mentioned above, we fixed each AE variant’s unique hyperparameters (eg, the sparsity term penalty strength β in the SAE). Otherwise, combining each model’s unique hyperparameters with the selected 4 hyperparameters would make the computation infeasible. To determine the optimal unique hyperparameter settings, we fine-tuned each AE variant using a standardized setup (learning rate=1E-3, optimizer=Adam, latent dimension: input dimension=0.15, and activation=Sigmoid) and fixed these optimal hyperparameter values in the subsequent experiments. To account for variations arising from the random initialization of neural network weights and data splitting, each of the 168 studied hyperparameter configurations was trained and evaluated using 5-fold cross-validation, with the average performance of the Euclidean distance-based k-NN on the AE-produced latent representations reported for the 2 prediction targets—AKI onset and 1-year mortality—across the 5 runs. The performance of the Euclidean distance-based k-NN on both the raw data and the data transformed by PCA, retaining 99% of the variance, served as baseline performance. We used *F*_1_-scores, area under the precision-recall curve (AUPRC), and area under the receiver operating characteristic curve (AUROC) as evaluation metrics. The k-NN model was evaluated with neighborhood sizes of 5, 10, 15, and 20, respectively, considering that the size of the retrieved similar patient cohort often varies based on different clinical needs (eg, the varying complexity of different diseases). We trained the models on each of the 5 data splits for up to 2000 epochs using an early stopping mechanism, meaning that training was stopped if the validation loss did not improve for more than 5 consecutive epochs.

We then evaluated the performance of using Mahalanobis distance as the distance measure for k-NN on latent representations, focusing on two key questions: (1) Does k-NN with Mahalanobis distance on latent representations outperform k-NN with Euclidean distance? (assessing the effectiveness of Mahalanobis distance), and (2) Is applying Mahalanobis distance-based k-NN on latent representations more effective than applying it directly to raw data? (assessing the effectiveness of using AEs for EHR data transformation).

We first selected the best-performing hyperparameter configuration for each AE variant that achieved the highest *F*_1_-score using Euclidean distance-based k-NN with a neighborhood size of 5 to transform the raw data into latent representations. Given that we varied latent dimensions during training and considering the potentially significant impact of different latent dimensions on Mahalanobis distance-based k-NN performance, which could obscure the actual characteristics of each AE variant, we also selected the best-performing hyperparameter configuration for each AE variant with latent-to-input dimension ratio fixed at 0.5 to transform the raw data into latent representations. Due to the significant computational cost of estimating the Mahalanobis distances, we randomly sampled 50% of the AE-transformed training and test datasets from each of the 5-fold data splits for evaluation on the KUMC dataset and only evaluated the performance with a neighborhood size of 5. Fixed random seeds were used to ensure the sampled data remained consistent across all AE variant evaluations. For the MCW dataset, we randomly sampled 70% of the data following the same procedure to ensure a comparable sample size to the sampled KUMC dataset.

### Statistical Analysis

One-tailed paired *t* test was used to assess whether one AE variant significantly outperformed the other with Euclidean-based k-NN across the 168 different hyperparameter configurations, with *P*<.01 considered statistically significant. Considering that in actual practice, neural network models are often fine-tuned to achieve optimal or near-optimal performance, we used an error bar plot to compare the average performance of the top 5 hyperparameter configurations for each AE variant, with Euclidean distance-based k-NN evaluated at neighborhood sizes of 5, 10, 15 and 20. This represents the upper performance bound of each AE variant in retrieving similar patients. We used box plots to visualize the impact of hyperparameter configurations on model performance. Each box plot shows the performance with Euclidean distance, where one hyperparameter of interest was fixed at a specific value while all other hyperparameters varied for each AE model.

To assess the generalizability of our findings from the KUMC dataset, we applied Spearman rank correlation to evaluate the relationship between model performance on the KUMC dataset and that on the MCW dataset. The Spearman rho (ρ) value was used to measure the strength and direction of the monotonic relationship between performances on the 2 datasets. A higher ρ indicates a stronger correlation, suggesting good generalizability.

### Ethical Considerations

All data were deidentified according to the “Safe Harbor” criteria outlined in the Health Insurance Portability and Accountability Act. The study was determined to be nonhuman participants research by the University of Florida Institutional Review Board, as it involved only pre-existing, deidentified patient records. The data access request was approved by the Greater Plains Collaborative Data Request Oversight Committee. This study was determined by the institutional review boards of the University of Florida, University of Pittsburgh Medical Center, and University of Missouri as nonhuman participant research because it only involved the collection of existing and deidentified patient medical data. Data use agreements have been executed with both the Greater Plains Collaborative and the University of Pittsburgh.

## Results

### Study Population

The final KUMC dataset encompassed 13,752 unique patients, while the MCW dataset encompassed 9568 patients. The AKI onset rates for the 2 datasets were 11.90% and 9.03%, respectively, and the 1-year mortality rates were 12.65% and 15.51%, respectively. The KUMC dataset contained 579 features, including 277 medications, 288 procedures, and 14 lab tests, while the MCW dataset contained 654 features, including 328 medications, 312 procedures, and 14 lab tests. The details of the 2 datasets are presented in Table 2.

**Table 2.** Statistics of the 2 datasets used in the study.

	KUMC^a^	MCW^b^
Cohort size	13,752	9568
Time window	January 1, 2016 to December 31, 2016	January 1, 2016 to December 31, 2016
AKI rates, n (%)	1636 (11.90)	890 (9.30)
1-year mortality rates, n (%)	1736 (12.65)	1484 (15.51)
Age (years), median (IQR)	61 (48-71)	61 (48-71)
Female, n (%)	6902 (50.19)	4772 (49.87)
Black race, n (%)	1856 (13.50)	2018 (21.09)
Days from admission to AKI^c^ onset (days), median (IQR)	7 (4-17)	5 (3-7)
Number of medication features	277	328
Number of procedure features	288	312
Number of lab test features	14	14

a KUMC: University of Kansas Medical Center.

b MCW: Medical College of Wisconsin.

c AKI: acute kidney injury.

### AE Performance With Euclidean Distance

**Figure 3.**
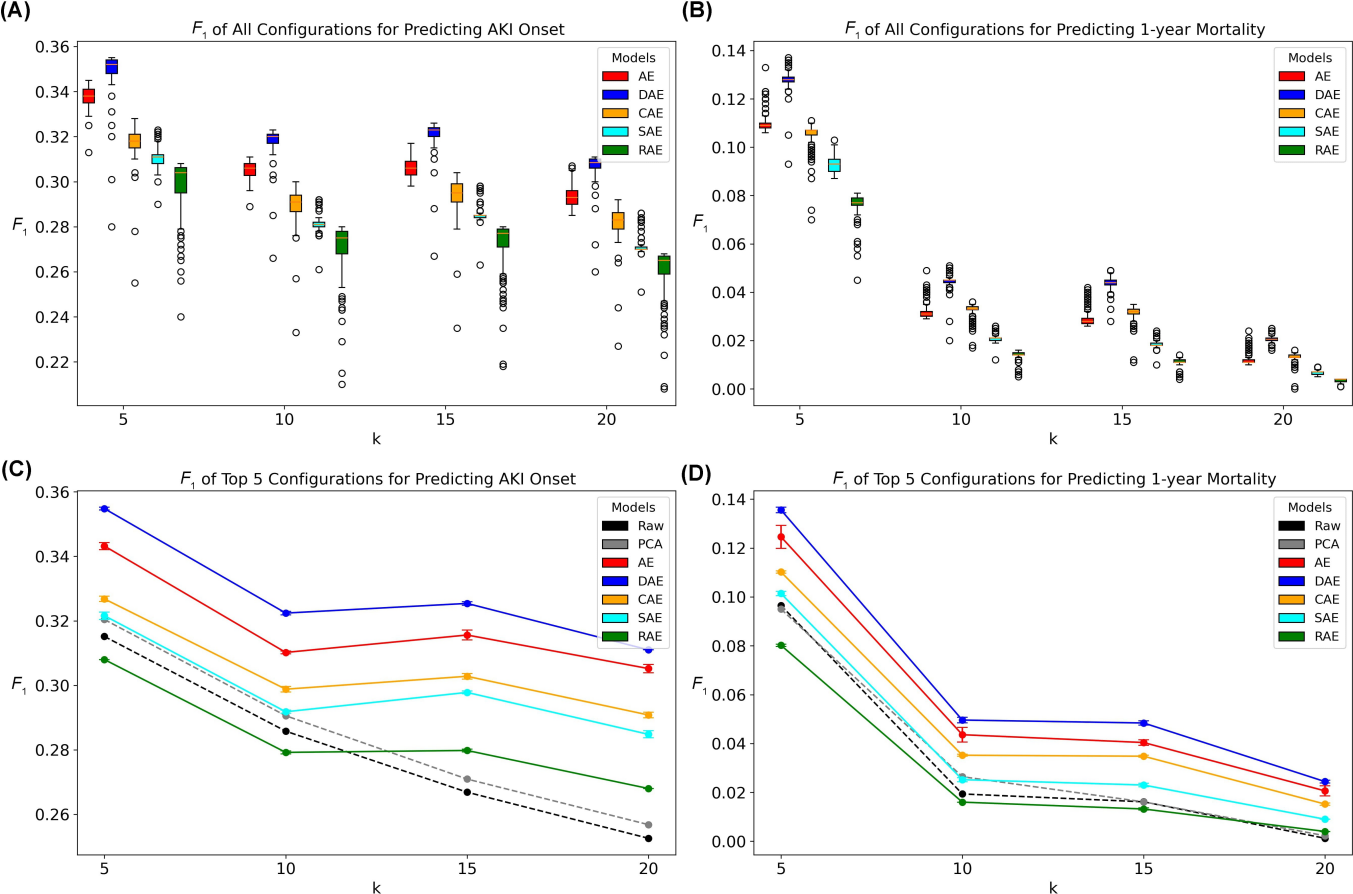
*F*_1_-scores of Euclidean-distance-based k-nearest neighbor models on the latent representations on the KUMC dataset. (****A**) ***F*_1_-scores of predicting AKI onset. Each box represents the k-nearest neighbor *F*_1_-scores with AE models trained with different hyperparameter configurations. (**B)** *F*_1_-scores of predicting 1-year mortality. (**C)** The mean *F*_1_-scores of the top 5 best AE hyperparameter configurations of predicting AKI onset. (**D)** The mean *F*_1_-scores of the top 5 best AE hyperparameter configurations of predicting 1-year mortality. AE: autoencoder; AKI: acute kidney injury; CAE: contractive autoencoder; DAE: denoising autoencoder; PCA: principal component analysis; RAE: robust autoencoder; SAE: sparse autoencoder.

The fine-tuned and fixed model-specific hyperparameters are provided in Multimedia Appendix 1. On the KUMC dataset, DAE consistently performed the best across both prediction targets (ie, AKI onset and 1-year mortality) and all k-NN neighborhood sizes (*P*<.001,Multimedia Appendix 2 and Multimedia Appendix 3), followed by vanilla AE and CAE (Figure 3, Figures S1 and S2 in Multimedia Appendix 4). The average performance of the top 5 hyperparameter configurations showed a similar trend, with DAE performing the best, followed by vanilla AE and CAE. For AKI onset prediction, CAE and SAE outperformed baseline models (ie, k-NN applied to the raw data and the PCA-transformed data) at *k*=15 and *k*=20 (Figure 3C) and performed comparably to the baseline models for 1-year mortality prediction (Figure 3D). The average best performance of RAE did not surpass that of the baseline models for 1-year mortality prediction (Figure 3D).

**Figure 4.**
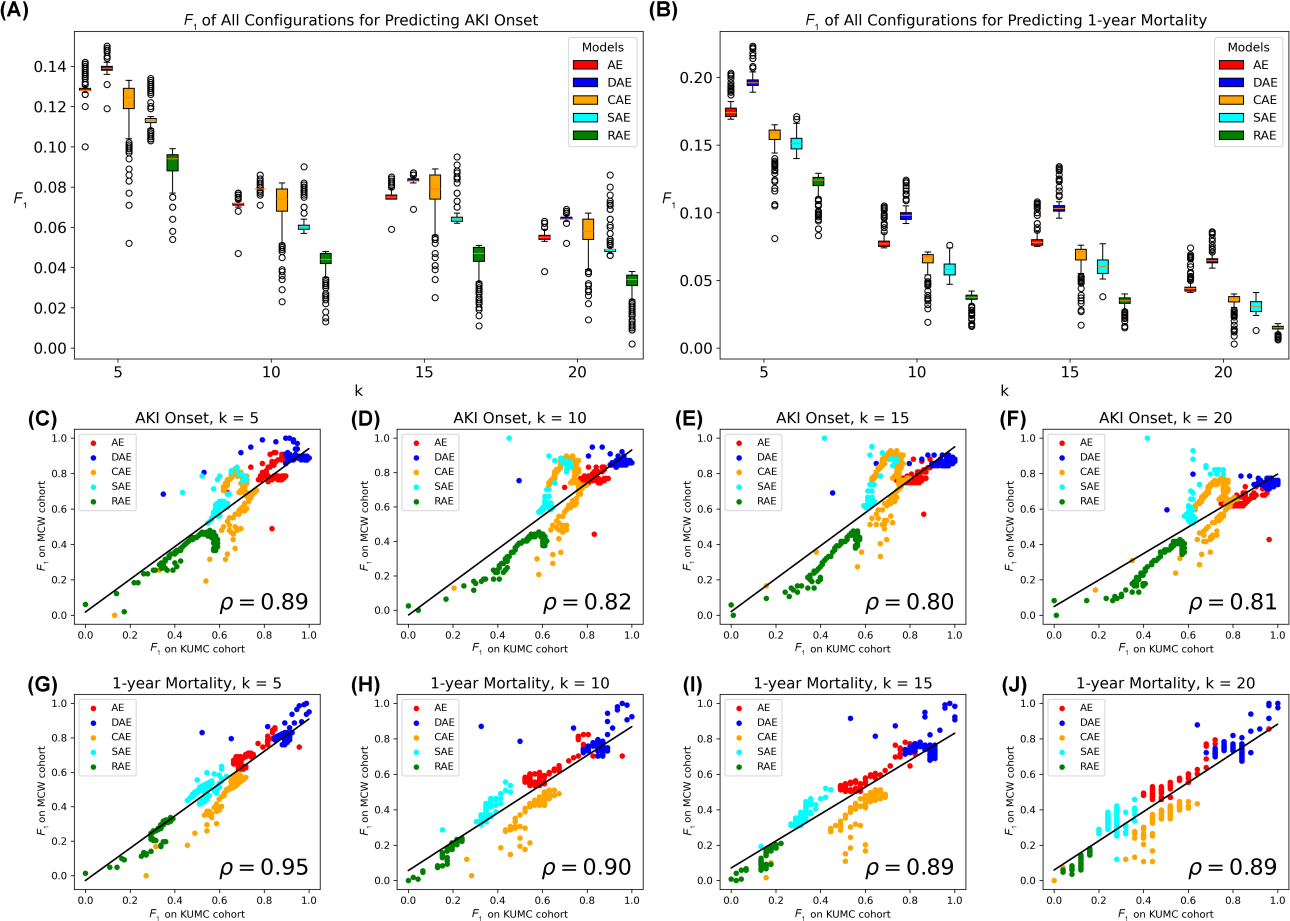
*F*_1_-scores of Euclidean-distance-based k-nearest neighbor models on the latent representations on the MCW dataset, and the linear correlation between the *F*_1_-scores on the KUMC and that on the MCW datasets. (**A)** *F*_1_-scores of predicting AKI onset. (**B)** *F*_1_-scores of predicting 1-year mortality.** (**C-F)**** Concordance in predicting AKI onset with varying neighborhood sizes. (**G-J)** Concordance in predicting 1-year mortality with varying neighborhood sizes. AE: autoencoder; AKI: acute kidney injury; CAE: contractive autoencoder; DAE: denoising autoencoder; KUMC: University of Kansas Medical Center; MCW: Medical College of Wisconsin; RAE: robust autoencoder; SAE: sparse autoencoder.

The performance of AE variants on the MCW dataset exhibited a clear resemblance to the results obtained on the KUMC dataset, with DAE consistently outperforming the other models (*P*<.001, Figures 4A and B). Correlation analysis showed that, across different hyperparameter settings, the *F*_1_-scores of AE variants were significantly correlated between both datasets, with *P*>.80 when predicting AKI onset (Figure 4C-F) and *P*>.89 when predicting 1-year mortality (Figure 4G-J). Similar results were also observed when using AUPRC and AUROC as metrics (Figures S3 and S4 in Multimedia Appendix 4).

### Impact of Hyperparameters on AE Performance

**Figure 5.**
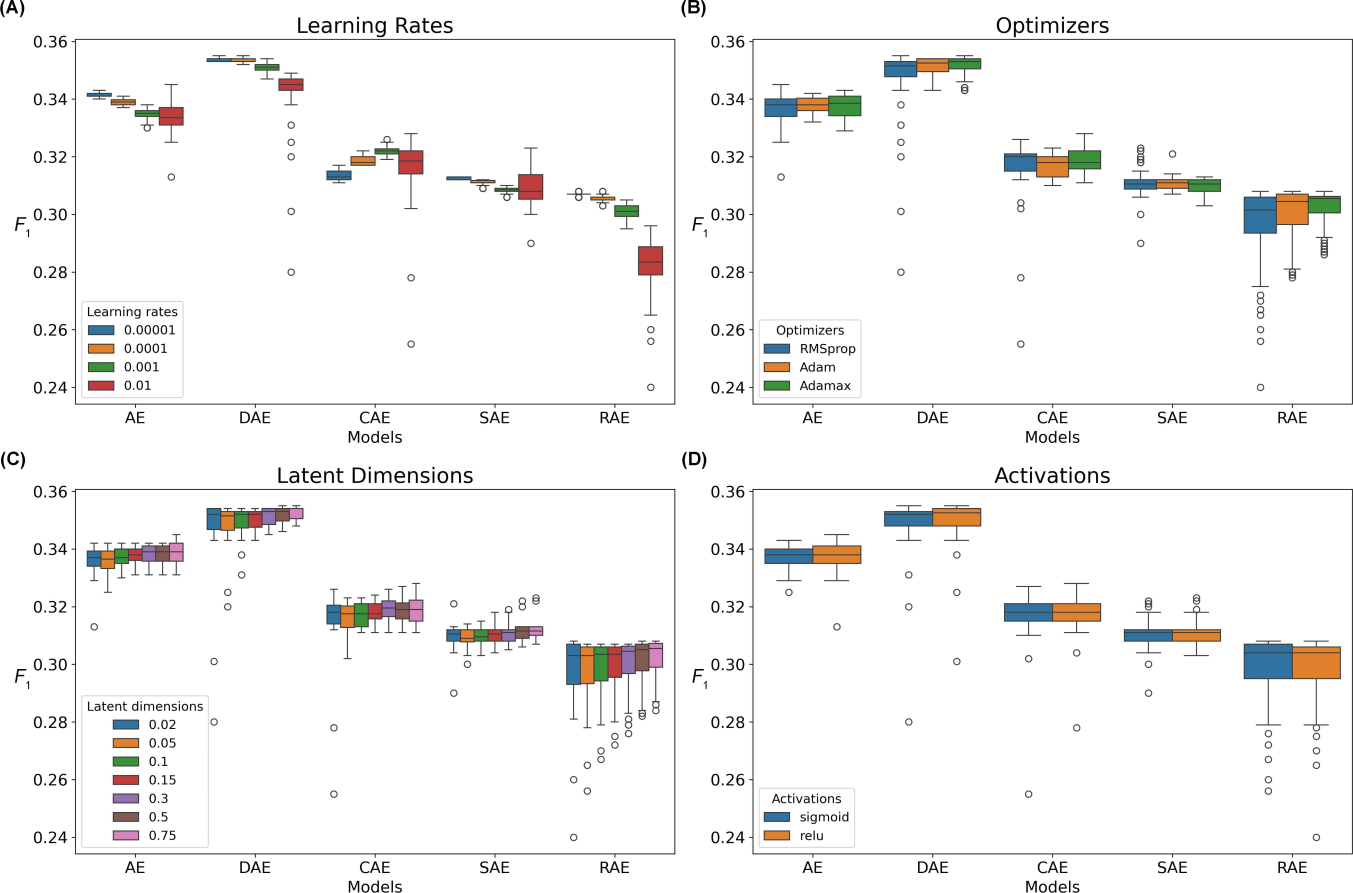
Impact of different AE hyperparameters on k-nearest neighbor model performance for predicting acute kidney injury onset on the University of Kansas Medical Center dataset. Each box plot shows the *F*_1_-scores with Euclidean distance and a neighborhood size of 5, when one hyperparameter was fixed, while varying all other hyperparameters for each AE model. (**A)** Learning rates; (**B)** Optimizers; (**C)** The ratio of latent representation dimension to input data dimension; (**D)** Activation functions. AE: autoencoder; CAE: contractive autoencoder; DAE: denoising autoencoder; RAE: robust autoencoder; SAE: sparse autoencoder.

On the KUMC dataset, when the neighborhood size was 5 and AKI onset was the prediction target, we observed that different hyperparameter settings had varying impacts on model performance. For learning rates, smaller values led to better performance for vanilla AE, DAE, and RAE. For CAE, a moderate learning rate yielded better results. At a higher learning rate (1E-2), the variance in model performance increased, with the upper bound observed in CAE and SAE outperforming those of other learning rates (Figure 5A). In terms of the optimizer, Adamax resulted in a slightly higher lower bound of model performance, while the upper bound showed no significant differences across optimizers (Figure 5B). Similarly, for latent dimensionality, higher dimensions (latent dimension: input dimension=0.75) led to a higher lower bound of model performance, with no significant differences observed in the upper bound (Figure 5C). No significant differences were observed between sigmoid and rectified linear unit activations (Figure 5D). Highly similar hyperparameter trends were observed on the KUMC dataset with neighborhood sizes of 10, 15, and 20 using *F*_1_-scores as the metric (Figures S5-S7 in Multimedia Appendix 4), and with a neighborhood size of 5 using AUPRC and AUROC as metrics (Figures S8 and S9 in Multimedia Appendix 4), as well as on the MCW dataset with a neighborhood size of 5 (Figures S10-S12 in Multimedia Appendix 4).

### AE Performance With Mahalanobis Distance

When comparing the performance of Euclidean distance-based k-NN and Mahalanobis distance-based k-NN on the KUMC dataset, we found that Mahalanobis distance-based k-NN generally performed better than Euclidean distance, except in a few cases (eg, DAE+NCA in Table 3). These performance drops primarily occur in NCA and MLKR, while LMNN consistently outperforms the Euclidean distance. This conclusion can be well generalized to the performance when controlling the latent dimension ratio (latent-to-input dimension ratio=0.5, Table 4) and the results on the MCW dataset (Multimedia Appendices 5 and 6).

**Table 3.** *F*_1_-scores of k-nearest neighbors (k-NNs) with Euclidean and Mahalanobis distances on the latent representations produced by the best-performing hyperparameter configuration of each autoencoder (AE) variant on the University of Kansas Medical Center dataset. The values are presented as mean (SD) of the 5-fold cross-validation.

Model	Euclidean distance, mean (SD)	Mahalanobis distance, mean (SD)		
		LMNN^a^	NCA^b^	MLKR^c^
Raw	0.284 (0.032)	0.314 (0.030)	0.325 (0.070)	0.381 (0.023)
AE	0.330 (0.030)	0.345 (0.046)	0.336 (0.052)	0.333 (0.029)
DAE^d^	0.364 (0.027)	0.385 (0.025)	0.354 (0.033)	0.374 (0.031)
CAE^e^	0.338 (0.028)	0.356 (0.035)	0.340 (0.037)	0.371 (0.038)
SAE^f^	0.332 (0.032)	0.354 (0.028)	0.323 (0.021)	0.325 (0.034)
RAE^g^	0.344 (0.025)	0.412 (0.037)	0.375 (0.029)	0.401 (0.012)

a LMNN: large margin nearest neighbor.

b NCA: neighborhood components analysis.

c MLKR: Metric Learning for Kernel Regression.

d DAE: denoising autoencoder.

e CAE: contractive autoencoder.

f SAE: sparse autoencoder.

g RAE: robust autoencoder.

When comparing the performance of Mahalanobis distance-based k-NN on latent representations versus directly on raw data, no consistent pattern was observed. On the KUMC dataset, in most cases, Mahalanobis distance-based k-NN on latent representations outperformed its performance on the raw data. However, there was no fixed pattern for the optimal combination of AE variants and Mahalanobis distance estimation algorithms. When latent dimensions were not controlled, the combination of RAE and all 3 investigated Mahalanobis distance algorithms achieved the best performance (Table 3). In contrast, when latent dimensions were controlled, the combination of DAE with LMNN and NCA and the combination of RAE with MLKR performed the best (Table 4). On the MCW dataset, limited cases showed that Mahalanobis distance-based k-NN on latent representations outperformed its application on the raw data, indicating that this pattern is data-dependent (Multimedia Appendices 5 and 6).

**Table 4.** *F*_1_-scores of k-nearest neighbors (k-NNs) with Euclidean and Mahalanobis distances on the latent representations produced by the best-performing hyperparameter configuration, constrained by a latent-to-input dimension ratio of 0.5 for each autoencoder (AE) variant, on the University of Kansas Medical Center (KUMC) dataset.

Model	Euclidean distance, mean (SD)	Mahalanobis distance, mean (SD)		
		LMNN^a^	NCA^b^	MLKR^c^
Raw	0.284 (0.032)	0.314 (0.030)	0.325 (0.070)	0.381 (0.023)
AE	0.320 (0.027)	0.368 (0.039)	0.330 (0.009)	0.337 (0.025)
DAE^d^	0.353 (0.025)	0.378 (0.032)	0.360 (0.016)	0.348 (0.021)
CAE^e^	0.337 (0.016)	0.345 (0.023)	0.342 (0.029)	0.363 (0.028)
SAE^f^	0.323 (0.040)	0.357 (0.051)	0.332 (0.039)	0.317 (0.035)
RAE^g^	0.291 (0.030)	0.327 (0.048)	0.359 (0.029)	0.396 (0.032)

a LMNN: large margin nearest neighbor.

b NCA: neighborhood components analysis.

c MLKR: Metric Learning for Kernel Regression.

d DAE: denoising autoencoder.

e CAE: contractive autoencoder.

f SAE: sparse autoencoder.

g RAE: robust autoencoder.

## Discussion

### Main Findings

This study makes significant contributions in three main areas: (1) it is the first to comprehensively evaluate the performance of different AEs specifically for EHR-based similar patient retrieval, providing critical insights to inform the design of AE-based patient representation learning models; (2) it is the first study to apply Mahalanobis distance to patient representations learned by AEs for similar patient retrieval, whereas previous studies have primarily relied on Euclidean distance; and (3) by establishing a fair and comprehensive evaluation framework, this study offers valuable guidance for AE model selection and hyperparameter tuning, contributing to the advancement of patient representation learning in EHR research.

Our findings indicate that DAE consistently outperformed other AEs, followed by vanilla AE and CAE, with RAE performing the worst. The superior performance of DAE likely stems from its mechanism of introducing noise into the original data during training, which encourages the model to prioritize encoding meaningful latent nonlinear relationships that are important for disease and outcome prediction, rather than focusing on noise. This process helps the model remain robust to noise, enabling more refined and abstracted patient representations, which improve the performance of the downstream k-NN model.

Other AE mechanisms, such as those in CAE and SAE, are also designed to produce effective representations and have performed well in regression and classification tasks in previous studies [17,29]. However, they were less effective than DAE in retrieving similar patients. For SAE, the assumed Bernoulli distribution over the latent representations may mismatch the continuous outputs of the hidden layer, conflicting with the need for sufficient active neurons to preserve key data information. This trade-off between enforcing sparsity and preserving key data information can significantly degrade downstream k-NN performance, especially on complex data. The RAE was originally designed for image classification tasks [28] and used an MCC loss rather than MSE to make the model more robust to outliers. However, this approach may not be well-suited to EHR data, which are often high-dimensional, sparse, noisy, and biased, resulting in its underperformance compared with other AE variants. Moreover, one of the prediction targets was AKI onset. Patients at high risk for developing AKI may show significant differences in lab results and medications compared with the general population. However, the MCC loss may weaken the encoding of this information in the latent representation, making the latent representations of high-risk patients with AKI less distinguishable from those in the normal population, which in turn leads to inaccuracies in similar patient retrieval (ie, higher false negative rates).

While both DAE and RAE are designed to enhance model robustness to noise, their underlying mechanisms differ significantly. DAE achieves this by explicitly injecting noise into the input data and training the model to “denoise” it, thereby encouraging the model to focus on capturing the intrinsic structure of the data. In contrast, RAE aims to improve robustness by making the model less sensitive to the input’s tail distribution, which reduces the influence of outliers. However, this approach may prevent the model from fully capturing the true data distribution. This fundamental difference could be a key factor contributing to the performance gap observed between DAE and RAE in the specific scenarios examined in this study.

Interestingly, while previous research suggests AE models are highly sensitive to hyperparameter configurations [24], our findings indicate that for the task of retrieving similar patients, only the learning rate significantly affected model performance. Specifically, smaller learning rates resulted in stronger lower-bound performance, as shown in Figure 5. However, larger learning rates (eg, 1E-2) could help the model escape local minima in certain situations, although not always guaranteed. Given the longer training times associated with smaller learning rates, we recommend using a moderate learning rate (eg, 1E-3 or 1E-4).

The impact of latent dimensions on model performance was minimal. Even with smaller latent dimensions (latent dimension to input dimension ratio=0.02), the latent representations remained expressive enough to ensure accurate patient similarity estimation. As shown in Figure 5, increasing the latent dimensions to accommodate additional information only slightly improved the model’s lower-bound performance. However, this conclusion is highly dataset-dependent, and other datasets may require larger latent dimensions to capture more information.

Regarding the application of Mahalanobis distance to latent representations, it outperformed Euclidean distance in most cases. Mahalanobis distance applies a low-dimensional linear transformation to map the representations into a space where the margin between different classes is maximized while representations of the same class are pulled closer together, thereby enhancing the discriminative power of the downstream k-NN model [41]. This result was expected, as Mahalanobis distance estimation treats latent representations as a separate dataset for learning appropriate linear feature transformations and distance measures. Compared with Euclidean distance, Mahalanobis distance is more likely to provide a more accurate estimation of vector similarity both within and between classes.

However, comparisons between Mahalanobis distance estimation algorithms on raw data and latent representations revealed that AE transformations of raw data did not always guarantee better performance. For example, AEs significantly improved the performance of Mahalanobis distance-based k-NN on the KUMC dataset (Tables3  4), but this improvement was limited on the MCW dataset (Multimedia Appendices 5 and 6). There can be several reasons behind this. First, differences in the characteristics of the datasets could play a major role. Variations in feature distributions, data sparsity, or sample size between the KUMC and MCW datasets may affect the effectiveness of AEs in learning meaningful latent representations. If the KUMC dataset contains clearer structure or more consistent patterns, AEs may be able to capture more relevant patient representations compared with the MCW dataset.

Additionally, the alignment between the AE-learned latent representations and the assumptions underlying Mahalanobis distance could also contribute to the observed differences. AEs are typically optimized for reconstruction rather than directly preserving class separability or the local neighborhood structure needed for effective distance-based retrieval. As a result, the learned representations may not always be well-suited for Mahalanobis distance algorithms, particularly if the AE fails to retain key relationships present in the raw data (eg mapping all data points into an indistinguishable cluster in the latent space). This suggests that whether this complex transformation process enhances model performance is highly data-dependent.

It is also important to note that Mahalanobis distance estimation algorithms tend to have higher computational complexity compared with Euclidean distance. For example, the loss function of LMNN is non-convex, requiring the use of semidefinite programming techniques to address this challenge [37]. Therefore, while the combination of “AE + Mahalanobis distance” can achieve optimal performance in certain cases, it is data-dependent and comes at the cost of increased computational complexity. To mitigate this burden, the dimensionality of the latent representation can be intentionally constrained, provided it maintains sufficient discriminative power for the downstream task.

### Limitations

This study only investigated swap noise for DAE; other types of noise, such as Gaussian noise, may lead to different behaviors. Additionally, we only examined AKI onset and 1-year mortality, so the models’ performance may differ for other prediction tasks. For example, other studies have shown that DAE outperforms vanilla AE and RAE on multiple datasets and tasks, though not in all cases [29]. Next, this study only compared AE variants trained in an unsupervised manner. Incorporating labels during training may help learn more effective latent representations compared with purely unsupervised approaches. Finally, while AEs can effectively capture complex patterns in high-dimensional clinical data, their latent representations are often difficult to interpret clinically, which may limit their utility in real-world decision-support settings. A disentangling framework should be further investigated and incorporated into the current AE model to enhance interpretability by isolating clinically meaningful latent factors, thereby facilitating more transparent integration into clinical decision support systems[42].

### Conclusions

In this study, we assessed the performance of 5 AE variants—vanilla AE, DAE, CAE, SAE, and RAE—on 2 real-world EHR datasets, focusing on retrieving similar patients for personalized clinical decision-making. The study also explored the impact of different hyperparameter configurations on AE variants. Our results presented three key findings: (1) DAE generally performed best in retrieving similar patients when paired with Euclidean distance (*P*<.001); (2) learning rates had the greatest impact on the performance of AE variants; and (3) applying Mahalanobis distance-based k-NN on latent representations can outperform Euclidean distance-based k-NN, although transforming raw data with AE variants did not always guarantee improved performance of Mahalanobis distance-based k-NN.

## Supplementary material

10.2196/68830 Multimedia Appendix 1 Model-specific hyperparameters fine-tuned and fixed for downstream experiments. In DAE, ρ denotes the amount of noise added to the data. In CAE, λ denotes the strength of the additional Frobenius norm penalty term. In SAE, ρ denotes the mean of the Bernoulli distribution, and β denotes the strength of the additional KL divergence penalty term. In RAE, σ denotes the variance of the Gaussian distribution, ρ denotes the mean of the Bernoulli distribution, β denotes the strength of the additional KL divergence penalty term, and λ denotes the strength of the weight decay. CAE: contractive autoencoder; RAE: robust autoencoder; SAE: sparse autoencoder.

10.2196/68830 Multimedia Appendix 2 *P* value tables showing the statistical significance of the *F*_1_-score differences between different AE variants across the 168 different hyperparameter configurations of predicting AKI onset on the KUMC dataset, using a one-tailed paired *t* test. *P*<.01 was considered statistically significant. Each entry represents the *P* value indicating whether the AE model in the row outperformed the AE model in the column.

10.2196/68830 Multimedia Appendix 3 *P* value tables showing the statistical significance of the *F*_1_-score differences between various AE variants across 168 different hyperparameter configurations for predicting 1-year mortality on the KUMC dataset.

10.2196/68830 Multimedia Appendix 4 Supplementary figures.

10.2196/68830 Multimedia Appendix 5 *F*_1_-scores of k-NNs with Euclidean distance and with Mahalanobis distance on the latent representations produced by the best-performing hyperparameter configuration, constrained by a latent-to-input dimension ratio of 0.5 for each AE variant, on the MCW dataset.

10.2196/68830 Multimedia Appendix 6 *F*_1_-scores of k-NNs with Euclidean distance and with Mahalanobis distance on the latent representations produced by the best-performing hyperparameter configuration of each AE variant on the MCW dataset.

## Abbreviations

AE: autoencoder
AKI: acute kidney injury
AUPRC: area under the precision-recall curve
AUROC: area under the receiver operating characteristic curve
CAE: contractive autoencoder
DAE: denoising autoencoder
EHR: electronic health record
k-NN: k-nearest neighbor
KL: Kullback-Leibler
LMNN: large margin nearest neighbor
MCC: maximum correntropy criterion
MCW: Medical College of Wisconsin
MLKR: Metric Learning for Kernel Regression
MSE: mean squared error
NCA: neighborhood components analysis
PCA: principal component analysis
RAE: robust autoencoder
SAE: sparse autoencoder
SCr: serum creatinine
