# Supplementary material for: Autoencoder-Based Representation Learning for Similar Patients Retrieval From Electronic Health Records: Comparative Study
Source: JMIR Med Inform. 2025 Jul 24;13:e68830. doi: 10.2196/68830 (PMC12289314; doi:10.2196/68830)
Supplement: Multimedia Appendix 1 [file medinform-v13-e68830-s001.docx]

| **Model** | **Model-specific Hyperparameters** |
| --- | --- |
| Vanilla AE | / |
| DAE | $\rho=0.1$ |
| CAE | $\lambda=1E-4$ |
| SAE | $\rho=0.05$,$\beta=3$ |
| RAE | $\sigma=1E-4$, $\rho=0.05$,$\beta=3$, $\lambda=0.03$ |
