# Supplementary material for: Autoencoder-Based Representation Learning for Similar Patients Retrieval From Electronic Health Records: Comparative Study
Source: JMIR Med Inform. 2025 Jul 24;13:e68830. doi: 10.2196/68830 (PMC12289314; doi:10.2196/68830)
Supplement: Multimedia Appendix 3 [file medinform-v13-e68830-s003.docx]

|  | **k = 5** | | | | | **k = 10** | | | | |
| --- | --- | --- | --- | --- | --- | --- | --- | --- | --- | --- |
|  | **AE** | **DAE** | **CAE** | **SAE** | **RAE** | **AE** | **DAE** | **CAE** | **SAE** | **RAE** |
| **AE** | / | 1.00 | <.001 | <.001 | <.001 | / | 1.00 | 0.87 | <.001 | <.001 |
| **DAE** | / | / | <.001 | <.001 | <.001 | / | / | <.001 | <.001 | <.001 |
| **CAE** | / | / | / | <.001 | <.001 | / | / | / | <.001 | <.001 |
| **SAE** | / | / | / | / | <.001 | / | / | / | / | <.001 |
| **RAE** | / | / | / | / | / | / | / | / | / | / |
|  | **k = 15** | | | | | **k = 20** | | | | |
| **AE** | / | 1.00 | 1.00 | <.001 | <.001 | / | 1.00 | 0.99 | <.001 | <.001 |
| **DAE** | / | / | <.001 | <.001 | <.001 | / | / | <.001 | <.001 | <.001 |
| **CAE** | / | / | / | <.001 | <.001 | / | / | / | <.001 | <.001 |
| **SAE** | / | / | / | / | <.001 | / | / | / | / | <.001 |
| **RAE** | / | / | / | / | / | / | / | / | / | / |
