# Supplementary material for: Autoencoder-Based Representation Learning for Similar Patients Retrieval From Electronic Health Records: Comparative Study
Source: JMIR Med Inform. 2025 Jul 24;13:e68830. doi: 10.2196/68830 (PMC12289314; doi:10.2196/68830)
Supplement: Multimedia Appendix 4 [file medinform-v13-e68830-s004.docx]

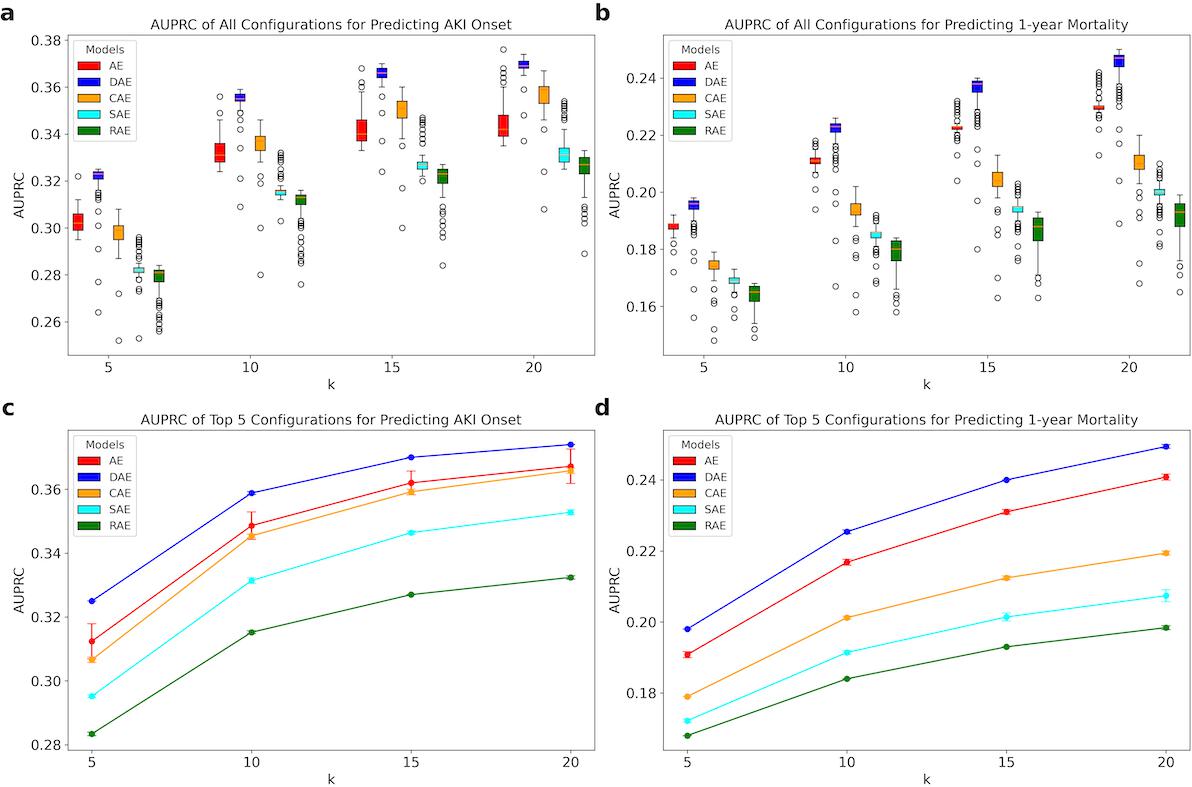


Figure S1. Area under the precision-recall curve (AUPRC) of Euclidean-distance-based k-NN models on the latent representations on the KUMC dataset. a AUPRC of predicting AKI onset. b AUPRC of predicting 1-year mortality. c Mean AUPRC of the top 5 best AE hyperparameter configurations of predicting AKI onset. d Mean AUPRC of the top 5 best AE hyperparameter configurations of predicting 1-year mortality.

[
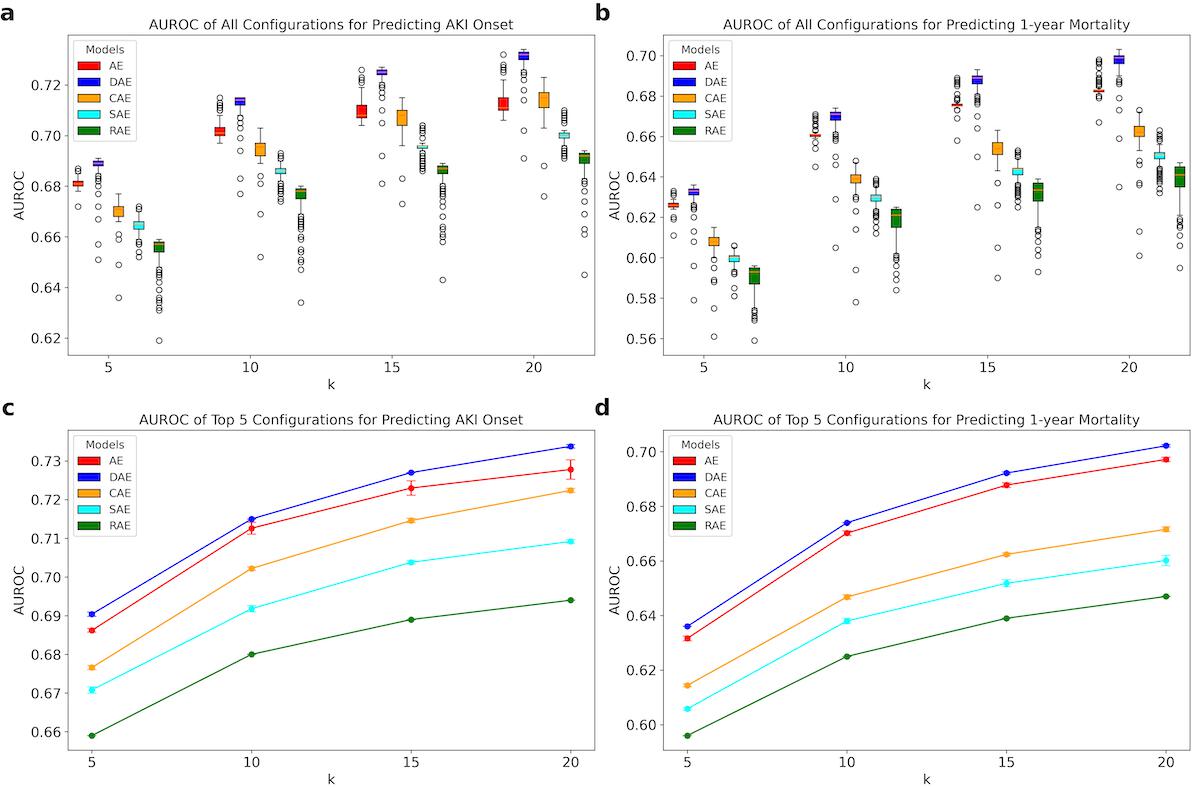
](https://jmir.kriyadocs.com/resources/jmir/medinform/68830/resources/416f72fd-2121-4958-a01c-7be484f9701c.png.JPEG)Figure S2. AUROC of Euclidean-distance-based k-NN models on the latent representations on the KUMC dataset. a AUROC of predicting AKI onset. b AUROC of predicting 1-year mortality. c Mean AUROC of the top 5 best AE hyperparameter configurations of predicting AKI onset. d Mean AUROC of the top 5 best AE hyperparameter configurations of predicting 1-year mortality.

[
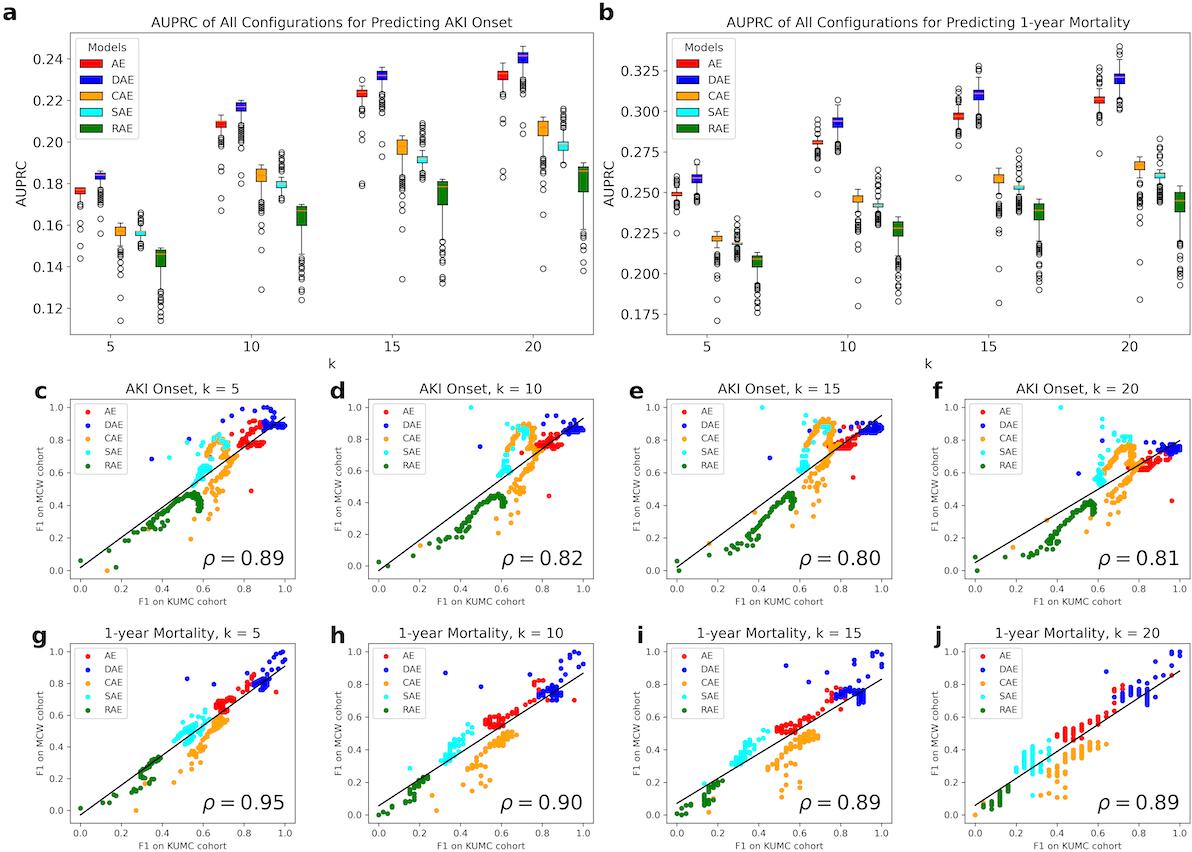
](https://jmir.kriyadocs.com/resources/jmir/medinform/68830/resources/f4315b6f-f144-47a4-ba96-c46efd600186.png.JPEG)Figure S3. AUPRC of Euclidean-distance-based k-NN models on the latent representations on the MCW dataset. a AUPRC of predicting AKI onset. b AUPRC of predicting 1-year mortality. c Mean AUPRC of the top 5 best AE hyperparameter configurations of predicting AKI onset. d Mean AUPRC of the top 5 best AE hyperparameter configurations of predicting 1-year mortality.

[
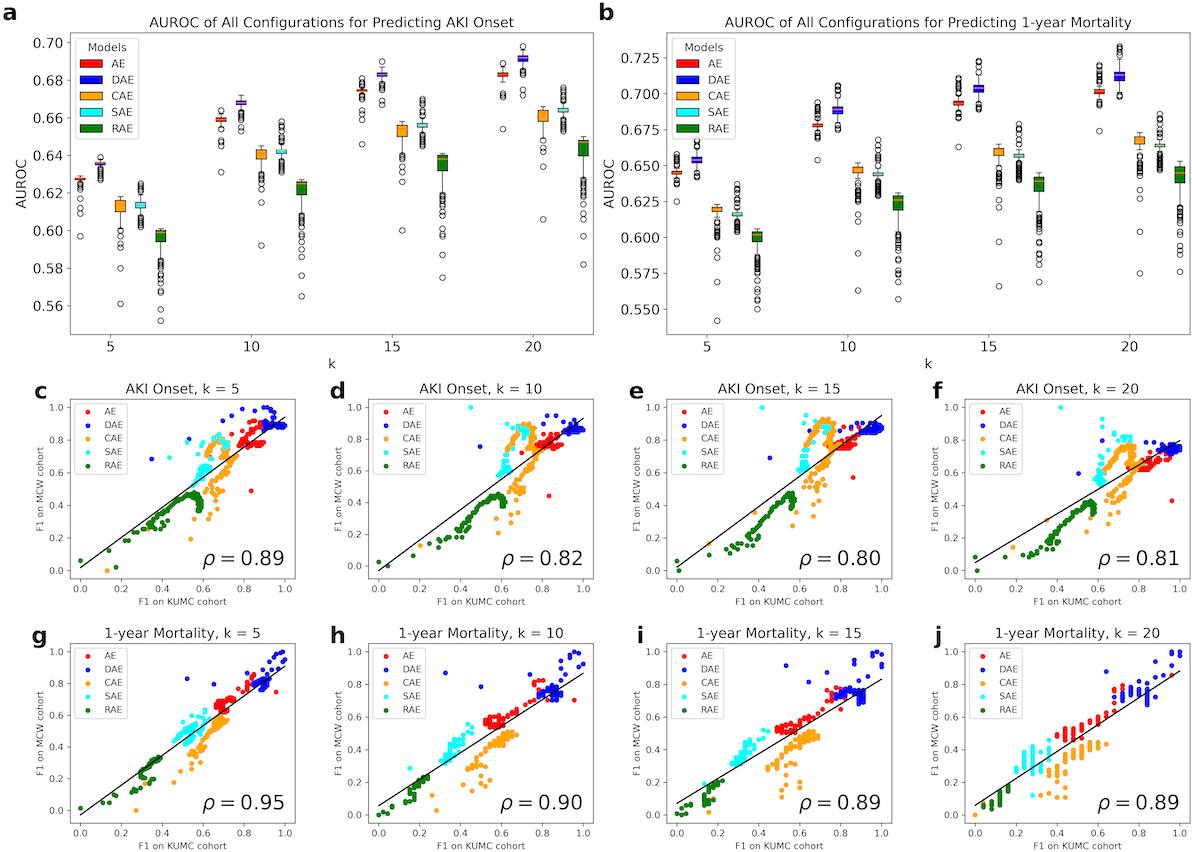
](https://jmir.kriyadocs.com/resources/jmir/medinform/68830/resources/819d8f5e-b630-4ef6-b50c-adfc43644920.png.JPEG)Figure S4. AUROC of Euclidean-distance-based k-NN models on the latent representations on the MCW dataset. a AUROC of predicting AKI onset. b AUROC of predicting 1-year mortality. c Mean AUROC of the top 5 best AE hyperparameter configurations of predicting AKI onset. d Mean AUROC of the top 5 best AE hyperparameter configurations of predicting 1-year mortality.

[
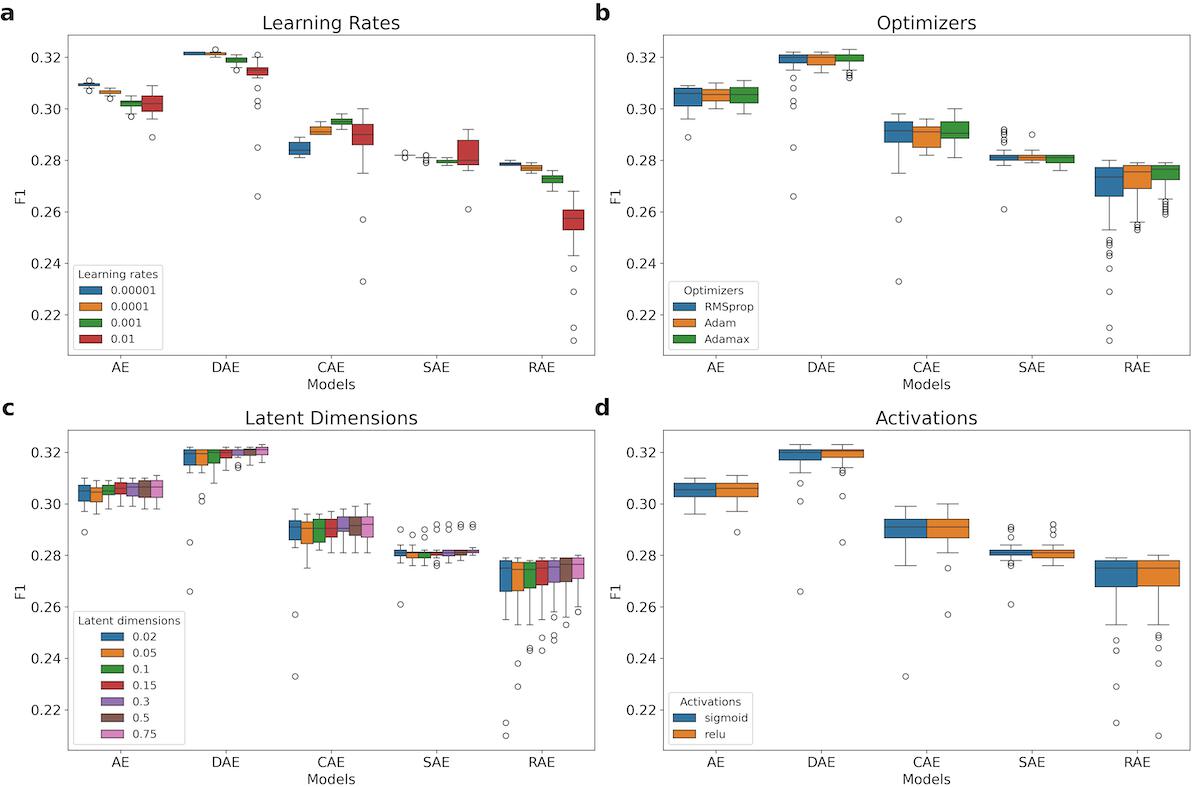
](https://jmir.kriyadocs.com/resources/jmir/medinform/68830/resources/e11e0b23-a424-4094-8d15-7c009fb6ffd4.png.JPEG)Figure S5. Effect of different AE hyperparameters on k-NN model performance for predicting AKI onset with a neighborhood size of 10 on the KUMC dataset with *F*_1_-scores as the metric.

[
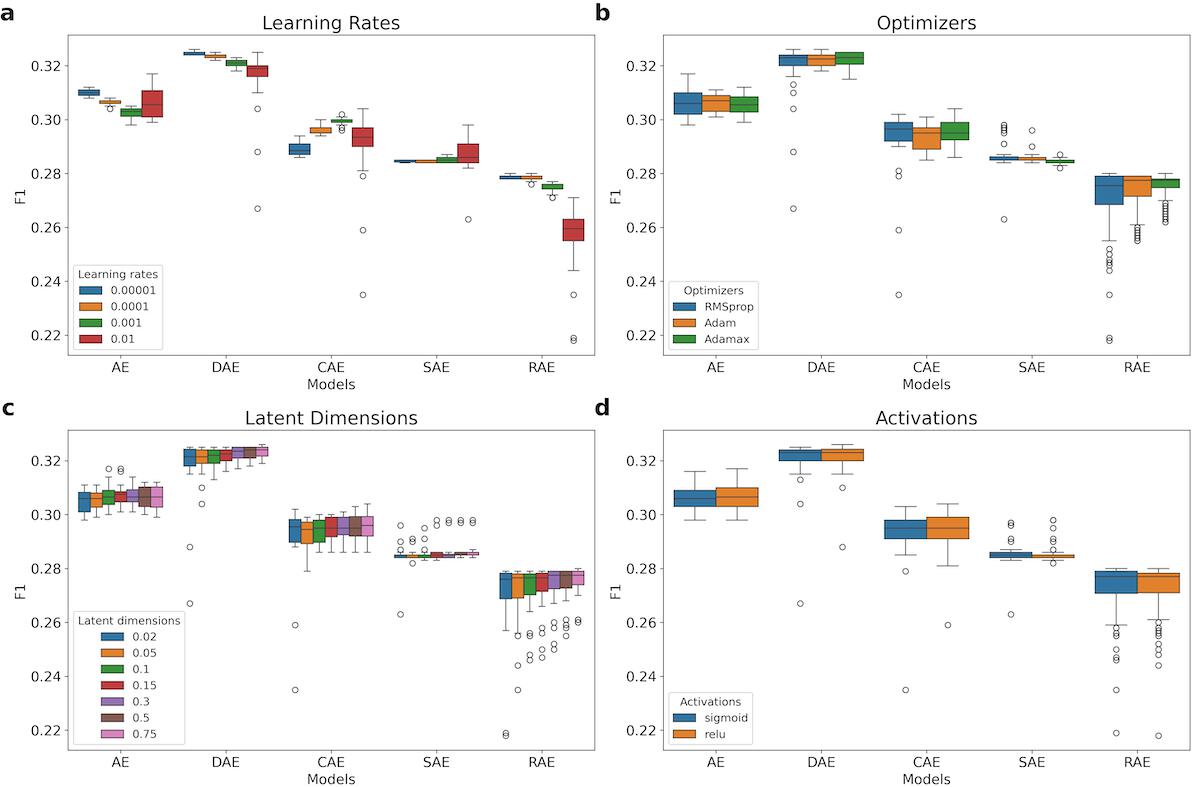
](https://jmir.kriyadocs.com/resources/jmir/medinform/68830/resources/8d759831-c254-40bb-83ac-3c250c07a2ae.png.JPEG)Figure S6. Effect of different AE hyperparameters on k-NN model performance for predicting AKI onset with a neighborhood size of 15 on the KUMC dataset with *F*_1_-scores as the metric.

[
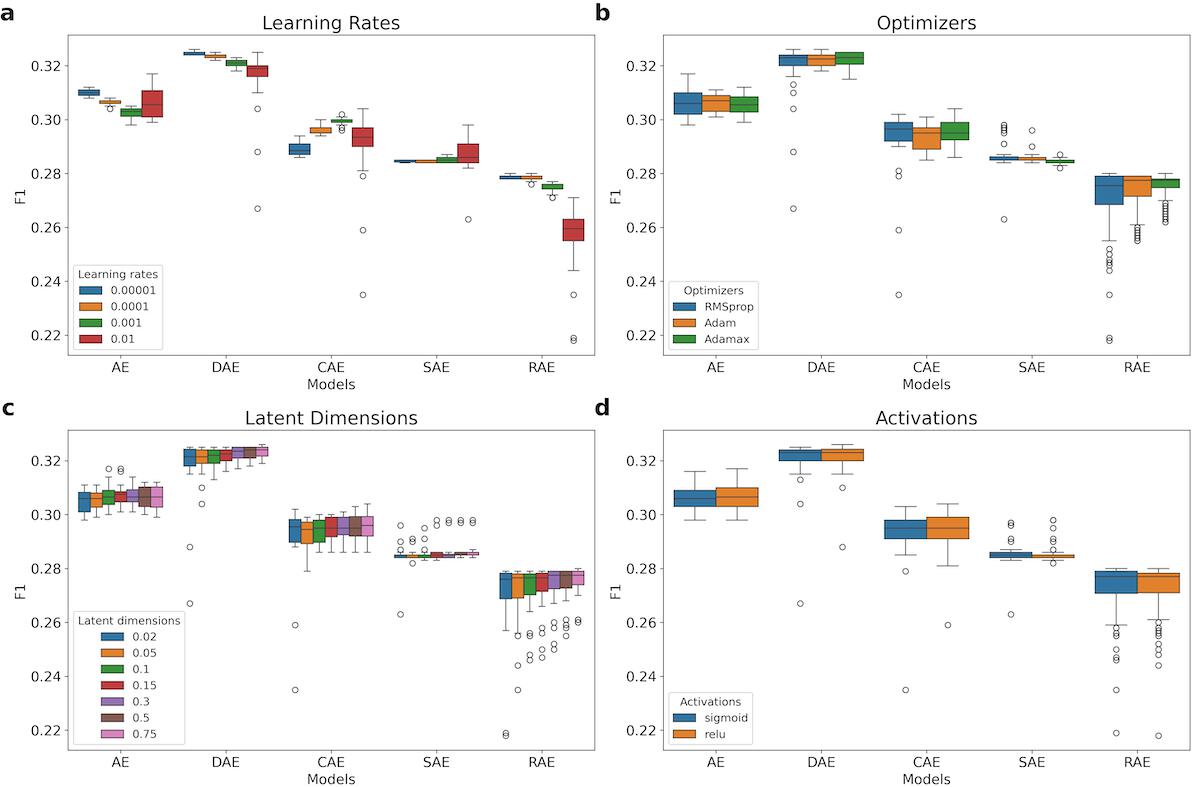
](https://jmir.kriyadocs.com/resources/jmir/medinform/68830/resources/b57dd8f1-d39c-4617-8c89-165826cfb4dc.png.JPEG)Figure S7. Effect of different AE hyperparameters on k-NN model performance for predicting AKI onset with a neighborhood size of 20 on the KUMC dataset with *F*_1_-scores as the metric.

[
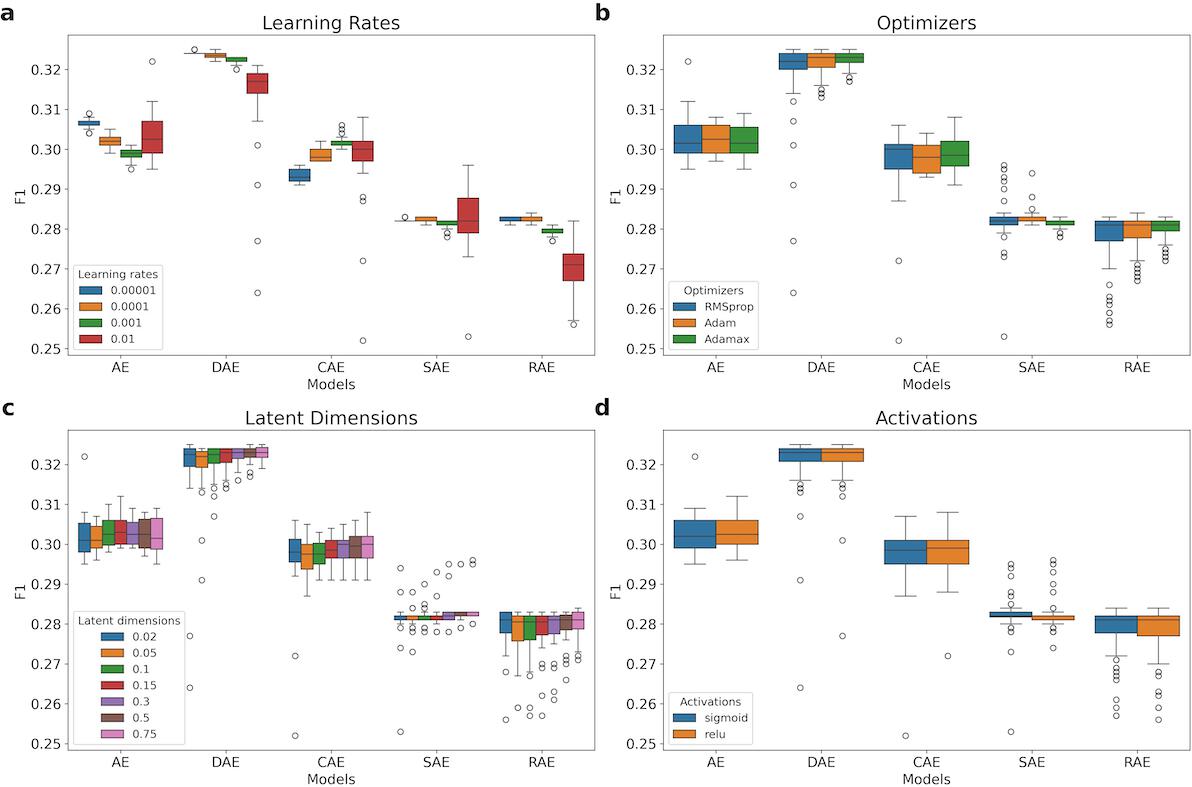
](https://jmir.kriyadocs.com/resources/jmir/medinform/68830/resources/a99aeef9-b1a3-4538-804f-73cc97a40238.png.JPEG)Figure S8. Effect of different AE hyperparameters on k-NN model performance for predicting AKI onset with a neighborhood size of 5 on the KUMC dataset with AUPRC as the metric.

[
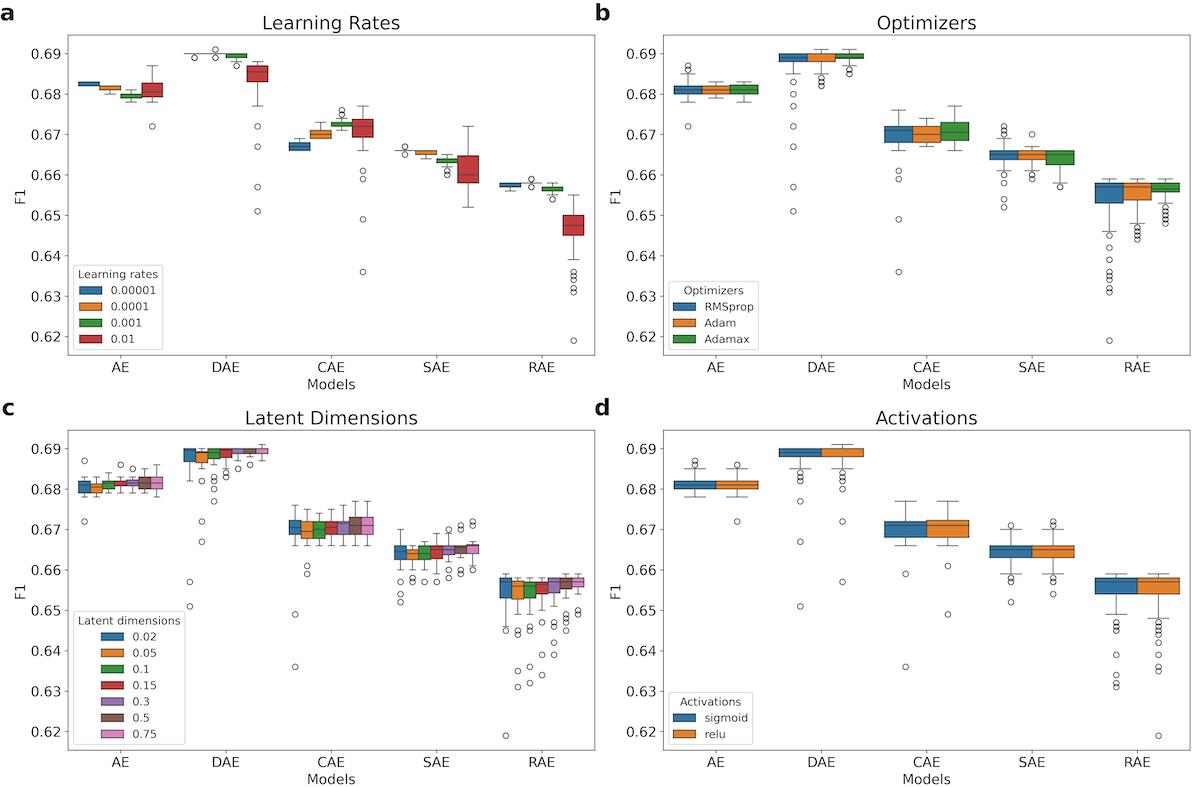
](https://jmir.kriyadocs.com/resources/jmir/medinform/68830/resources/7c99538b-7589-4ba5-8f75-6e984e0dcd8d.png.JPEG)Figure S9. Effect of different AE hyperparameters on k-NN model performance for predicting AKI onset with a neighborhood size of 5 on the KUMC dataset with AUROC as the metric.

[
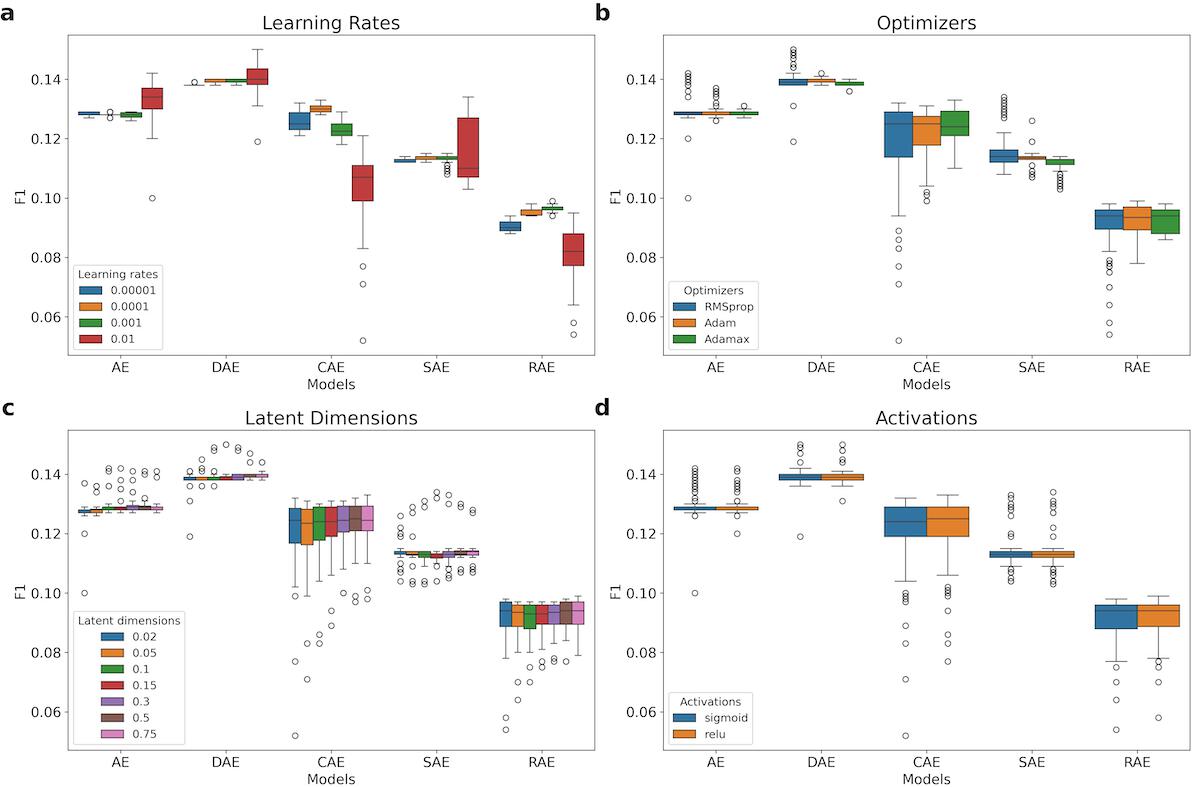
](https://jmir.kriyadocs.com/resources/jmir/medinform/68830/resources/0a66b79b-6bc0-4e74-9735-a32d14808945.png.JPEG)Figure S10. Effect of different AE hyperparameters on k-NN model performance for predicting AKI onset with a neighborhood size of 5 on the MCW dataset with *F*_1_-scores as the metric.

[
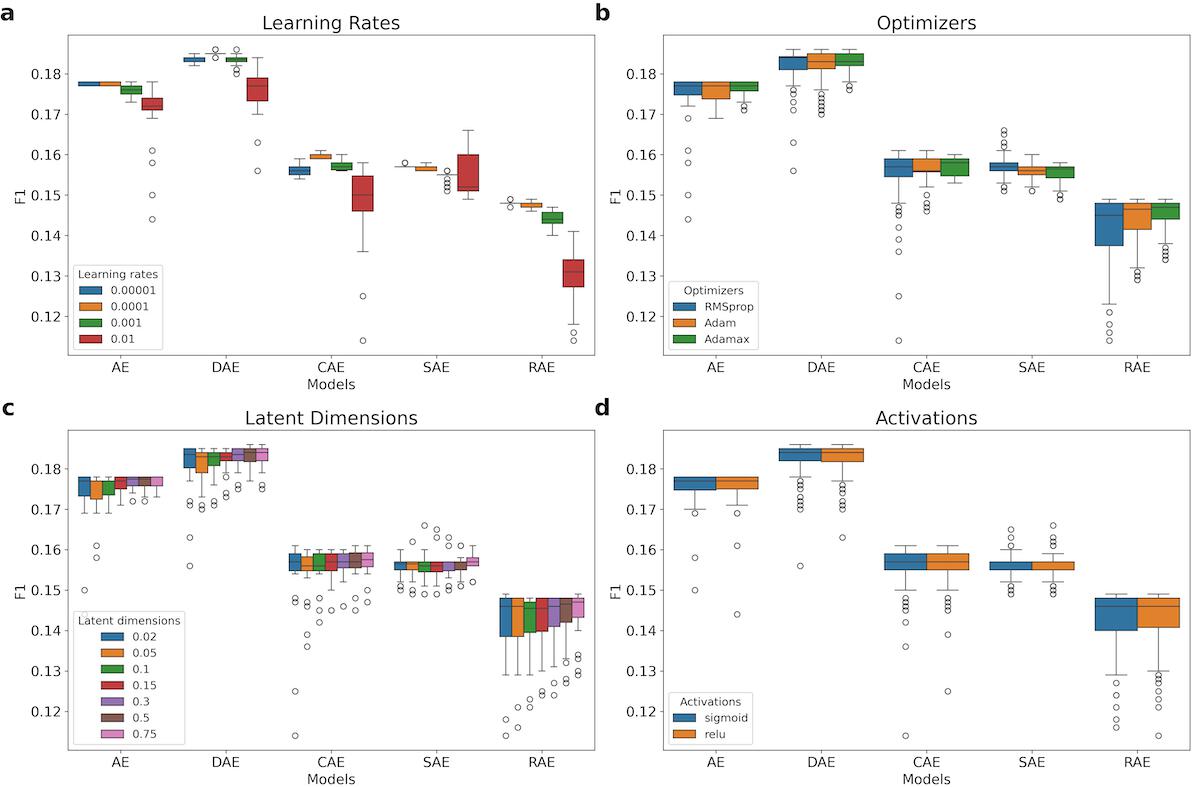
](https://jmir.kriyadocs.com/resources/jmir/medinform/68830/resources/f94ba372-c24c-4bea-80c8-ffd54a09bf51.png.JPEG)Figure S11. Effect of different AE hyperparameters on k-NN model performance for predicting AKI onset with a neighborhood size of 5 on the MCW dataset with AUPRC as the metric.

[
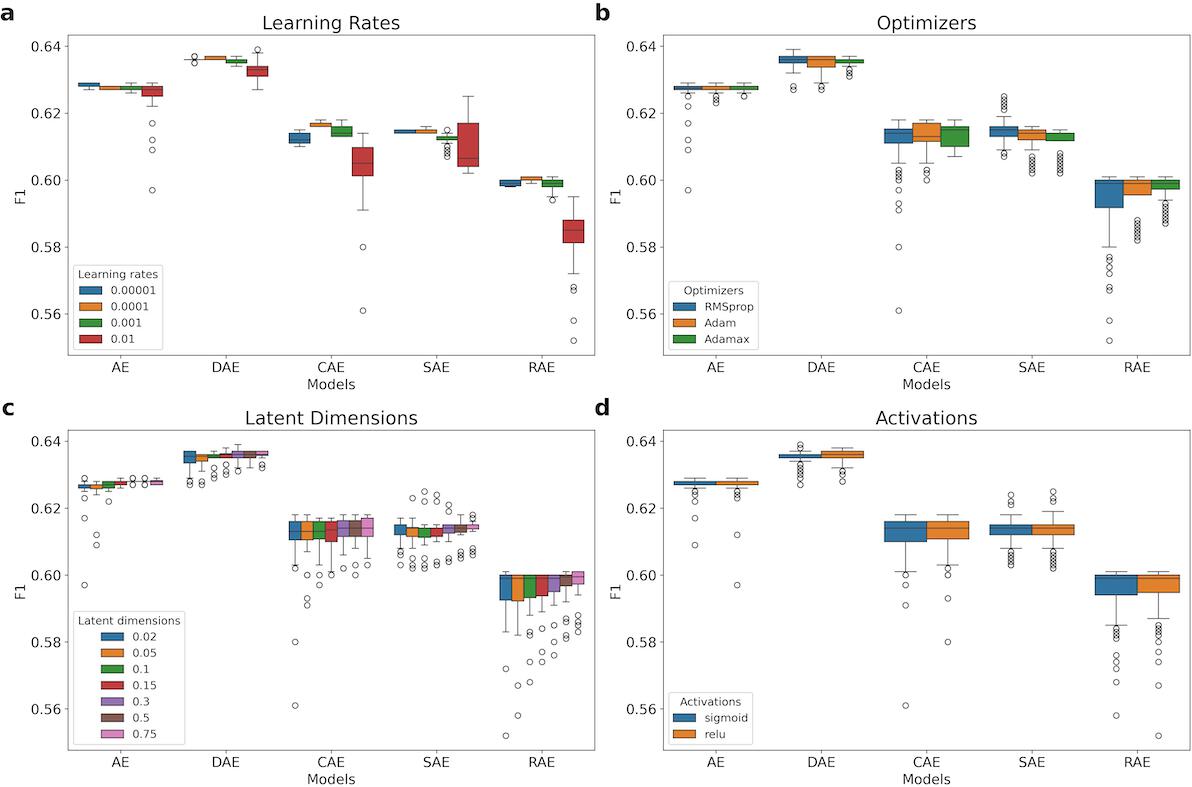
](https://jmir.kriyadocs.com/resources/jmir/medinform/68830/resources/c13bfc81-603a-43cf-86e5-bea249ae2203.png.JPEG)Figure S12. Effect of different AE hyperparameters on k-NN model performance for predicting AKI onset with a neighborhood size of 5 on the MCW dataset with AUROC as the metric.
