# Supplementary material for: Autoencoder-Based Representation Learning for Similar Patients Retrieval From Electronic Health Records: Comparative Study
Source: JMIR Med Inform. 2025 Jul 24;13:e68830. doi: 10.2196/68830 (PMC12289314; doi:10.2196/68830)
Supplement: Multimedia Appendix 5 [file medinform-v13-e68830-s005.docx]

| Model | Euclidean distance | Mahalanobison distance | | |
| --- | --- | --- | --- | --- |
|  |  | LMNN | NCA | MLKR |
| Raw | 0.095 (0.015) | **0.266 (0.030)** | **0.172 (0.043)** | 0.194 (0.038) |
| AE | 0.111 (0.034) | 0.154 (0.015) | 0.127 (0.042) | 0.134 (0.050) |
| DAE | 0.116 (0.027) | 0.154 (0.042) | 0.147 (0.025) | 0.148 (0.059) |
| CAE | **0.186 (0.037)** | 0.153 (0.041) | 0.164 (0.079) | 0.181 (0.019) |
| SAE | 0.106 (0.022) | 0.088 (0.037) | 0.090 (0.026) | 0.110 (0.034) |
| RAE | 0.101 (0.025) | 0.220 (0.039) | 0.133 (0.062) | **0.194 (0.033)** |
