# Supplementary material for: Autoencoder-Based Representation Learning for Similar Patients Retrieval From Electronic Health Records: Comparative Study
Source: JMIR Med Inform. 2025 Jul 24;13:e68830. doi: 10.2196/68830 (PMC12289314; doi:10.2196/68830)
Supplement: Multimedia Appendix 6 [file medinform-v13-e68830-s006.docx]

| Model | Euclidean distance | Mahalanobison distance | | |
| --- | --- | --- | --- | --- |
|  |  | LMNN | NCA | MLKR |
| Raw | 0.095 (0.015) | **0.266 (0.030)** | 0.172 (0.043) | 0.194 (0.038) |
| AE | 0.100 (0.015) | 0.112 (0.027) | 0.112 (0.050) | 0.112 (0.030) |
| DAE | 0.110 (0.017) | 0.199 (0.029) | 0.138 (0.034) | 0.158 (0.037) |
| CAE | **0.165 (0.045)** | 0.152 (0.036) | **0.185 (0.053)** | 0.175 (0.056) |
| SAE | 0.106 (0.057) | 0.124 (0.058) | 0.126 (0.062) | 0.111 (0.038) |
| RAE | 0.089 (0.026) | 0.206 (0.042) | 0.124 (0.048) | **0.217 (0.047)** |
